# Evolutionary conservation of early mesoderm specification by mechanotransduction in Bilateria

**DOI:** 10.1038/ncomms3821

**Published:** 2013-11-27

**Authors:** Thibaut Brunet, Adrien Bouclet, Padra Ahmadi, Démosthène Mitrossilis, Benjamin Driquez, Anne-Christine Brunet, Laurent Henry, Fanny Serman, Gaëlle Béalle, Christine Ménager, Frédéric Dumas-Bouchiat, Dominique Givord, Constantin Yanicostas, Damien Le-Roy, Nora M. Dempsey, Anne Plessis, Emmanuel Farge

**Affiliations:** 1 Mechanics and Genetics of Embryonic Development Group, PSL Research University, CNRS, UMR168, Inserm, Institut Curie, 11 rue Pierre et Marie Curie, 75005 Paris, France; 2 Physico-Chemistry of Electrolytes and Colloïds, CNRS UMR 7195—UPMC—ESPCI, 2 place Jussieu, 75005 Paris, France; 3 Univ. Grenoble Alpes, Inst NEEL, F-38042 Grenoble, France; 4 CNRS, Inst NEEL, F-38042 Grenoble, France; 5 Zebrafish Models of Neurodegenerative Disorders, Inserm, Hôpital Robert Debré, U676 Paris, France; 6 Institut Jacques Monod, CNRS, UMR 7592, Univ Paris Diderot, Sorbonne Paris Cité, F-75205 Paris, France; 7 These authors contributed equally to the work; 8 Present address: Developmental Biology Unit, European Molecular Biology Laboratory, D-69117 Heidelberg, Germany

## Abstract

The modulation of developmental biochemical pathways by mechanical cues is an emerging feature of animal development, but its evolutionary origins have not been explored. Here we show that a common mechanosensitive pathway involving β-catenin specifies early mesodermal identity at gastrulation in zebrafish and *Drosophila*. Mechanical strains developed by zebrafish epiboly and *Drosophila* mesoderm invagination trigger the phosphorylation of β-catenin–tyrosine-667. This leads to the release of β-catenin into the cytoplasm and nucleus, where it triggers and maintains, respectively, the expression of zebrafish *brachyury* orthologue *notail* and of *Drosophila* Twist, both crucial transcription factors for early mesoderm identity. The role of the β-catenin mechanosensitive pathway in mesoderm identity has been conserved over the large evolutionary distance separating zebrafish and *Drosophila*. This suggests mesoderm mechanical induction dating back to at least the last bilaterian common ancestor more than 570 million years ago, the period during which mesoderm is thought to have emerged.

## Abstract (web summary)

Mechanical cues can induce morphogenetic processes during development. Here the authors show that mechanical changes during embryonic development in both zebrafish and *Drosophila* lead to nuclear localization of β-catenin, which regulates genes required for early mesoderm development in both species.

## Graphical abstract

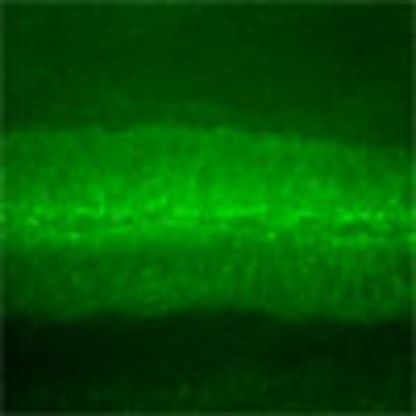

Gastrulation is a near-universal developmental step in eumetazoan embryonic development, which simultaneously involves the initiation of morphogenetic movements and important modulation of cell fate and identity^1^. It therefore requires intimate association of the mechanical and the molecular aspects of development. One of the proposed mechanisms involves feedback of mechanical signals to developmental transduction pathways and gene expression, a phenomenon known to occur in cell culture^2^,^3^,^4^ and in different developmental contexts^5^,^6^,^7^,^8^,^9^,^10^,^11^,^12^.

For instance, mechanical cues associated with the morphogenetic movements of convergent extension were suggested to regulate the apical stabilization of myosin-II (Myo-II) in ectoderm cell junctions in the *Drosophila* embryo, thereby reinforcing the tissue’s mechanical integrity in response to the initiation of embryonic morphogenetic movements^12^. Associated mechanical cues were also shown to vitally determine the anterior endoderm differentiation of *Drosophila* embryos^13^. Furthermore, the mechanical strains developed in the mesoderm by Snail-regulated active fluctuations of cell apex size were proposed to activate the Fog pathway that activates RhoGEF2-dependent apical submembranar stabilization of Myo-II, thereby triggering mesoderm invagination^14^. However, evolutionary conservation of the role of mechanotransduction in the development across species has not been explored so far.

Here we report a common mechanosensitive β-catenin (β-cat) pathway contributing critically to the induction of mesodermal cell fate during the gastrulation of zebrafish and *Drosophila*, two bilaterian species that diverged more than 570 million years ago. We find that the onset of morphogenetic movements in zebrafish embryos (epiboly) and in *Drosophila* embryos (mesoderm invagination) triggers the mechanotransductive activation of the phosphorylation of the Y667-β-cat site that impairs its interaction with E-cadherins. This leads to its release from the junctions to the nucleus, in the specifically strained mesodermal tissue of the gastrulating embryos. We find, as a consequence, mechanical induction of mesoderm-specific β-cat target genes expression in the strained mesodermal tissue of both species: *brachyury* (*bra*) ortholog *notail* (*ntl*) *in* zebrafish and Twist in *Drosophila*. This is, to our knowledge, the first report of an evolutionarily conserved mechanosensitive pathway having a major role in the developmental establishment of the body plan, in this case in early mesoderm specification. The search for a common biochemical pathway leading to mesoderm formation across Bilateria remains an open question of evolutionary developmental biology^1^,^15^, especially owing to the divergent case of the protostome superphylum Ecdysozoa, for which there has been no report of a role for β-cat in early mesoderm specification^16^. Our results suggest a common mechanotransductive origin of mesoderm emergence for Bilateria and is the first demonstration of a role for β-cat in early mesoderm determination in an ecdysozoan (*Drosophila melanogaster*)^1^,^15^,^16^,^17^.

## Results

### Nuclear translocation of β-cat in zebrafish embryos

**Figure 1:**
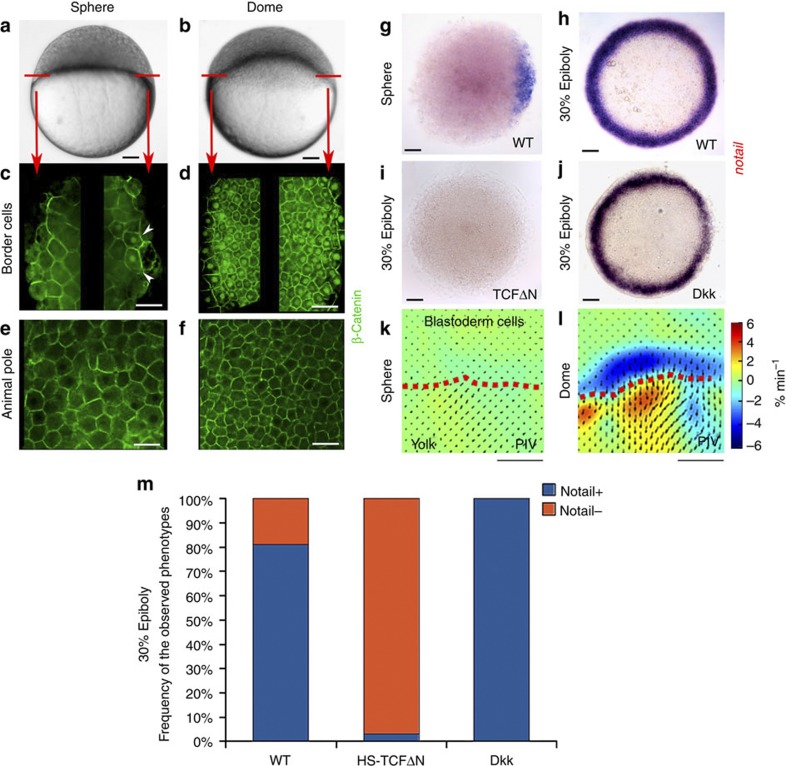
***ntl*** **expression is β-cat dependent but Wnt independent in the zebrafish embryo mesoderm at the onset of epiboly**. (**a**) Zebrafish embryos at sphere stage (4 hpf). (**b**) Dome-stage zebrafish embryos at the onset of epiboly (4.3 hpf). (**c**) β-cat labelling, margin cells (left) and dorsal pole (right, white arrows: nuclear β-cat) at sphere. (**d**) β-cat labelling around the margin (left and right) at dome (ubiquitous nuclear β-cat). (**e**) β-cat labelling at animal pole (upper pole) at sphere stage. (**f**) β-cat labelling at animal pole (upper pole) at dome stage. Scale bar, 20 μm (white bars). (**g**) *ntl* expression initiation in dorsal pole at sphere. (**h**) *ntl* expression initiation in margin zone at 30% epiboly (79/79). (**i**) *ntl* labelling at 30% epiboly in heat-shocked HS-TcfΔN-GFP embryos (26/26). (**j**) *ntl* labelling in *dkk*-injected embryos (8/8 controls, 8/8 *dkk*), as well as in heat-shocked HS:Dkk-GFP embryos, at 30% epiboly (14/14 WT siblings, 7/7 HS:Dkk-GFP). (**k**) PIV analysis with no significant cell deformation at sphere. (**l**) PIV analysis with marginal cell dilation at dome, coinciding with epiboly initiation. (**m**) Quantification of *ntl*-expressing embryos in HS-TcfΔN (*n*=35) and Dkk (*n*=15) compared with WT (*n*=37) at 50% epiboly in zebrafish. *P*<2.2E−16 by the *χ*^2^-test. All experiments were replicated two times. Scale bar, 100 μm (black and white bars).

In the early development of the zebrafish *Danio rerio*, nuclear translocation of β-cat occurs in two steps. The first step, starting at 128 cells and lasting until sphere stage (4 h post fertilization (hpf)), is under maternal gene control and defines the dorsal organizer by nuclear labelling restricted to a few cells (Fig. 1a,c,e, sphere stage, white arrowheads)^18^. In a second step, after epiboly has started, β-cat nuclear translocation expands to encompass the entire marginal zone^19^. By performing β-cat immunostainings on zebrafish embryos, we found that marginal β-cat nuclear translocation initiates during the first 20 min of the epiboly morphogenetic movement (Fig. 1b,d,f, dome stage).

The full circumference of the marginal zone out of the dorsal pole gives rise to the mesoderm in zebrafish^20^. In *Xenopus* and mice, β-cat has been shown to be necessary for early *bra* expression leading to mesoderm induction^21^,^22^. So far, it has been unclear whether β-cat was necessary to establish the expression of the *bra* orthologue *ntl* in zebrafish. β-cat being the co-transcription factor of Tcf, we used a heat-shock-inducible Tcf-dominant-negative-GFP (HS-TcfΔN) transgenic strain to evaluate this possibility^23^. In green fluorescent protein (GFP)-positive heat-shocked transgenic embryos, *ntl* expression is totally undetectable by *in situ* hybridization at 30% (4.7 hpf, Fig. 1h,i,m) and 50% (5.3 hpf, Supplementary Fig. S1a,b) epiboly. Consistently, in wild-type (WT) fish *ntl* expression was observed in cells characterized by nuclear β-cat only, indicating that *ntl* transcription is a cell autonomous response to β-cat nuclear translocation (Supplementary Fig. S1c). β-cat transcriptional activity is thus necessary for initiation of *ntl* expression in zebrafish, suggesting that *ntl* expression is under the control of Wnt ligands that are known to activate β-cat transcription-dependent events^24^.

Strikingly, injection of the inhibitor of the Wnt receptors *dkk* or overexpression of a HS:Dkk-GFP transgene did not result in the inhibition of *ntl* expression at 30% epiboly (Fig. 1j), whereas it did at the later 50% epiboly stage^25^. Nor did it prevent the initiation of β-cat nuclear translocation in margin cells at dome stage, although it did at the later 30% epiboly and subsequent stages (Supplementary Fig. S2a–c). The initiation of marginal nuclear β-cat and *ntl* expression is thus Wnt independent.

### Mechanically induced nuclear β-cat by epiboly

Nuclear translocation of β-cat has been shown to be inducible by mechanical forces in *Drosophila melanogaster* embryos^9^,^13^ and in mammalian cells^11^,^26^,^27^. As the onset of marginal β-cat nuclear translocation coincides with the first epiboly movements in zebrafish, we tested the hypothesis that nuclear translocation of β-cat at the margin of the zebrafish embryo is induced by the mechanical signals associated with epiboly movements. We first performed Particle-Image Velocimetry (PIV) analysis of FM-464-stained zebrafish embryos to test whether marginal tissue undergoes specific deformation during epiboly and indeed found the marginal zone to undergo local dilation of the tissue of the order of −4% min^−1^ at the onset of epiboly (Fig. 1k,l), in agreement with yolk contractile force pulling margin cells in epiboly^28^.

**Figure 2:**
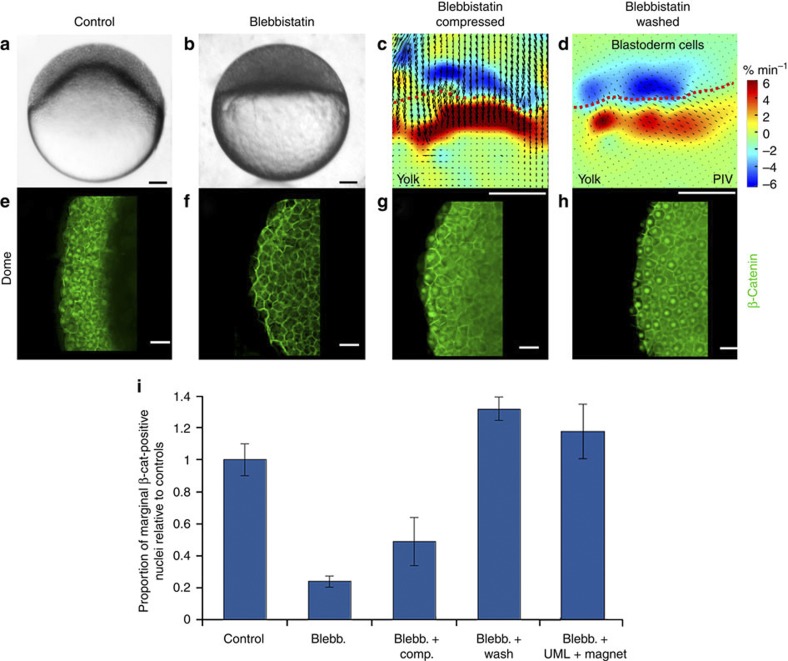
Mechanical induction of nuclear translocation of β-cat in the zebrafish embryo mesoderm. (**a**) 4.8 hpf embryos at 30% epiboly. (**b**) 4.8 hpf embryos blocked at sphere after treatment with blebbistatin. (**c**) Blebbistatin-treated embryo compression and the resumption of epiboly movements and marginal cell dilation. (**d**) Blebbistatin washing and resumption of epiboly movements and marginal cell dilation. Deformations are assessed by PIV analysis. Note that velocity fields differ between **c** and **d**, but the dilations of the marginal cells in blue are the same. Scale bar, 100 μm (black and white bar). (**e**) β-cat labelling around the margin in dome to 5.7 hpf non-treated 50% epiboly embryos. (**f**) β-cat labelling around the margin in blebbistatin-treated embryos (nuclear in the dorsal pole only, see Supplementary Fig. S4a). (**g**) β-cat labelling around the margin after global deformation of blebbistatin-treated embryos. (**h**) β-cat labelling after blebbistatin washing upon resumption of endogenous movements. Scale bar, 20 μm (white bar). (**i**) Quantification of marginal β-cat-positive nuclei in controls (*n*=16), blebbistatin-treated (*n*=22), blebbistatin-treated and globally compressed (*n*=16), blebbistatin-treated and washed (*n*=10), and blebbistatin treated embryos with epiboly rescue by magnetic forces (*n*=17). Differences between control and blebbistatin-treated embryos, and between treated embryos and rescued embryos, are statistically significant according to Mann–Whitney’s exact test (*P*<0.001). Error bars are s.d. Note that ectopic positive nuclei in blebbistatin-treated and compressed individuals (Supplementary Fig. S5c) are not taken into account in this quantification.

We thus tested whether such deformation was necessary for β-cat nuclear translocation around the margin. We found that blebbistatin, a specific non-muscle myo-II inhibitor^29^, can prevent zebrafish epiboly if treatment is started at the sphere stage (Fig. 2a,b), as is known of the microtubule-depolymerizing drug nocodazole^30^. Despite acting on different targets, both compounds were found to almost completely (80%) disrupt marginal β-cat nuclear translocation (Fig. 2e,f,i and Supplementary Fig. S3a,b,g). Consistently, the dorsal centre of β-cat nuclear translocation established before treatment was unaffected by both drugs (Supplementary Fig. S4a,b).

We then tested whether exogenous deformation of epiboly-inhibited embryos could rescue β-cat nuclear translocation. A soft uniaxial global compression of 35 μm applied during 20 min (see Methods) led to margin cell deformation quantitatively mimicking the dome-stage endogenous deformation of 4% min^−1^ (Fig. 2c), and induced β-cat nuclear translocation in a ubiquitous manner in epiboly-inhibited embryos (Fig. 2g and Supplementary Figs S3c and S5a–c,e–g). This resulted in a partial but significant 50% rescue of the positive nuclei proportion, if only the margin is considered (Fig. 2i and Supplementary Fig. S3g). To exclude any permissive role of drug treatment in β-cat mechanically induced nuclear translocation, we performed compression of oblong-stage non-treated embryos having not yet initiated epiboly. In non-compressed oblong-stage embryos, no nuclear translocation of β-cat is observed around the margin out of the dorsal pole (Fig. 1c,e and Supplementary Fig. S5i). β-cat nuclear translocation in the margin cells was also triggered at this pre-epiboly stage by compression in those non-treated embryos, thereby excluding any permissive role of drug treatment in mechanically induced β-cat nuclear translocation (Supplementary Fig. S5j–l).

To test whether β-cat nuclear translocation can be mechanically rescued by the strains developed owing to endogenous epiboly morphogenetic movements, we first took advantage of the fact that epiboly could be rescued within 5 min by washing away blebbistatin. PIV analysis confirmed that the WT −4% min^−1^ value of endogenous dilation characteristic of the dome shape change initiating epiboly was quantitatively restored in blebbistatin-treated and washed individuals (Fig. 2d). We observed a complete rescue of nuclear translocation around the deformed marginal zone only when the endogenous movements resumed (Fig. 2h,i and Supplementary Fig. S5d,h).

**Figure 3:**
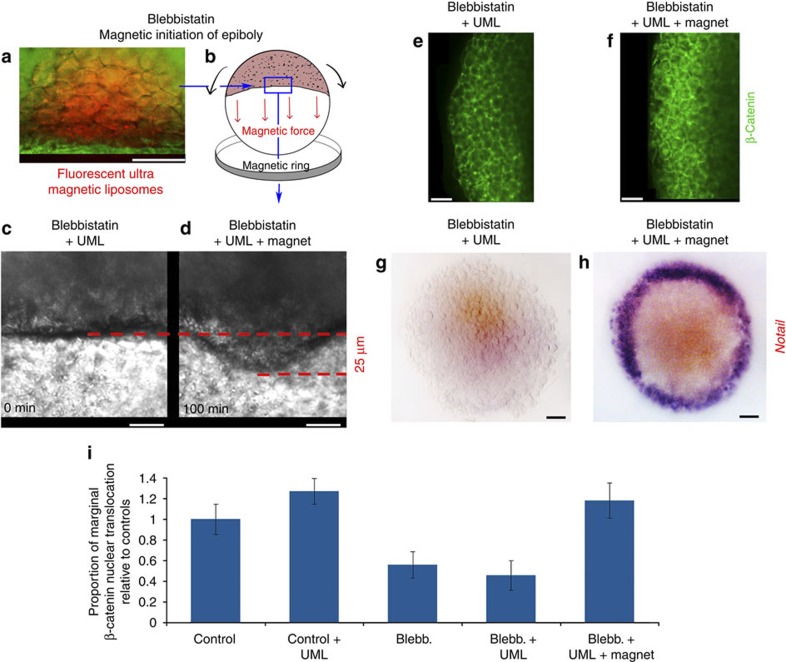
**Nuclear translocation of β-cat and of** ***ntl*** **expression by magnetically induced mimicking of the onset of epiboly**. (**a**) Embryos injected with UML (in red, green counter colour in transmission, surface lateral view). (**b**) Magnetic force applied to magnetically loaded margin cells by the ring micromagnet. (**c**) Margin tissue in blebbistatin-treated UML-injected embryos at time zero of magnetic field application, (**d**) and after 100 min of application. In the two later cases, the loss of resolution due to the ring micromagnet setup impaired PIV analysis (see Methods). (**e**) Labelling of β-cat in blebbistatin-treated UML-injected embryos in the absence of ring micromagnet. (**f**) Labelling of β-cat in blebbistatin-treated UML-injected embryos after exposure to the ring micromagnet (see Supplementary Fig. S9b for UML-injected embryo controls and Fig. 2i for quantification). (**g**) *ntl* Labelling in blebbistatin-treated UML-injected embryos in the absence of ring micromagnet (representative of *n*=12 embryos on *n*=15 injected embryos). (**h**) *ntl* Labelling after the ring micromagnet applied forces to UML-injected embryos (see Supplementary Fig. S9b for UML-injected embryo controls and Fig. 4e for quantification). (**i**) Quantification of nuclear translocation of β-cat in controls (*n*=5), UML-injected controls (*n*=5), blebbistatin-treated embryos (*n*=35), blebbistatin UML-injected embryos (*n*=4) and blebbistatin UML-injected embryos submitted to the ring micromagnet (*n*=17). All data are characterized by *P*<0.001 using Mann–Whitney’s exact test. Error bars are s.d. All experiments were replicated at least two times. Scale bars, 100 μm (black bars) and 20 μm (white bars).

Furthermore, we developed a new method of deformation by injecting blebbistatin-treated zebrafish embryos with ultramagnetic liposomes (UMLs) and exposing them to a permanent magnet ring positioned 400 μm below the border cells (see Methods). With this method, we rescued front epiboly movements in local margin cells, with a mean value of 0.25±0.06 μm min^−1^ (*n*=4). This is comparable with the value of 0.22±0.04 μm min^−1^ (*n*=5), which is characteristic of the onset of the first hour of the endogenous margin cell front movement at dome stage (Fig. 3a–d). We observed a complete rescue of nuclear translocation around the deformed marginal zone after the rescue of front movements in such border cells (Fig. 3e,f,i and Supplementary Fig. S9a).

We found that the magnetic loading of a quarter of the embryo by injection at a four-cell stage led to the nuclear translocation of β-cat in magnetically loaded cells, and at a short distance from magnetically loaded cells (Supplementary Fig. S6a,b). Some individual margin cells that were not loaded with magnetic liposomes effectively also showed β-cat nuclear translocation (Supplementary Fig. S6a). These cells can be as distant as three cells away from magnetically loaded cells, indicating that mechanical cues lead to non-cell-autonomous nuclear translocation of β-cat (Supplementary Fig. S6c).

Together, these results demonstrate that nuclear translocation of β-cat is highly sensitive to non-cell-autonomous mechanical activation in margin cells, which are specifically deformed by the morphogenetic movement of epiboly initiation.

### Mechanical induction of *ntl* expression by epiboly

**Figure 4:**
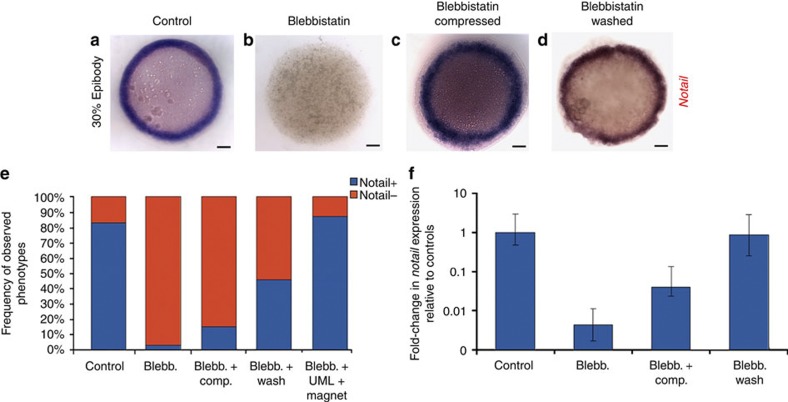
**Mechanical induction of** ***ntl*** **expression in the zebrafish embryo mesoderm**. (**a**) *ntl* Expression in control embryos at the germ ring stage of 30% to 50% epiboly (5.7 hpf). (**b**) *ntl* labelling in blebbistatin-treated embryos at the same stage, (**c**) in blebbistatin-treated embryos globally compressed at the same stage (**d**) and after blebbistatin washing at 4.8 and 5.7 hpf, when washed embryos morphologically reach 30–50% epiboly. Scale bar, 100 μm (black bar). (**e**) Quantification of the number of embryos showing *in situ ntl* expression in controls (*n*=97), blebbistatin-treated (*n*=95), blebbistatin-treated compressed (*n*=73), blebbistatin-treated and washed (*n*=24), and blebbistatin-treated embryos with epiboly rescue by magnetic forces (*n*=8). Differences between control and blebbistatin-treated embryos, and between treated and rescued embryos, are statistically significant according to the *χ*^2^-test (*P*<0.001). (**f**) Quantitative reverse-transcription PCR quantification of *ntl* expression in controls, blebbistatin-treated, blebbistatin-treated compressed, blebbistatin-treated and washed embryos (each reaction realized in technical triplicates). Differences between control and blebbistatin-treated embryos, and between treated and rescued embryos, are statistically significant according to the Student’s *t*-test (*P*<0.001). Error bars are s.d. All experiments were replicated two times.

We then explored the target of β-cat *ntl* expression in the margin cells of epiboly-inhibited embryos and compared this with mechanically and epiboly-rescued embryos. In epiboly-inhibited embryos, *ntl* expression displayed the following pattern. At the sphere stage, immediately after treatment, unipolar expression at the dorsal pole can be detected (Supplementary Fig. S4c,d). This corresponds to the maternally defined, *goosecoid* (*gsc*)-expressing part of the dorsal organizing centre (the future prechordal plate) specified by the maternally determined first wave of β-cat nuclear translocation (Fig. 1c) that transiently expresses *ntl*^31^. In WT embryos, *ntl* expression later spreads to the entire marginal zone and gets excluded from this *gsc+* part of the organizer^32^,^33^. In treated embryos, when control siblings reach 30% epiboly, *ntl* expression similarly vanishes from the *gsc*+ organizer cells but does not spread over the margin and thus becomes undetectable by *in situ* hybridization (Fig. 4a,b,e and Supplementary Fig. S3d,e,h). On the other hand, *gsc* expression remains readily detectable in the organizer of treated embryos (Supplementary Fig. S7a–d). Such selective clearance of *ntl* expression from the dorsal pole shows that treated embryos are not affected by a general developmental delay.

We first checked that the *ntl* expression default is secondary to a failure in β-cat transcriptional activity using inhibitors of GSK3β. We treated embryos submitted to blebbistatin and nocodazole with either nonspecific (LiCl) or highly specific (alsterpaullone, 1-azakenpaullone) inhibitors of GSK3β known to activate the β-cat pathway, by impairing the GSK3β-dependent cytoplasmic β-cat degradation and thus allowing its nuclear translocation^32^. We found that all of them efficiently rescued *ntl* expression at the margin of epiboly-inhibited embryos (Supplementary Fig. S8a–j). Interestingly, *ntl* expression is rescued only at the margin and not ubiquitously, which is consistent with a previous report of ubiquitous Wnt8 overexpression that did not result in *ntl* ectopic expression^34^. This indicates the existence of a β-cat-independent pre-pattern that restricts the competence for β-cat-induced *ntl* expression to marginal cells.

We then tested whether exogenously applied mechanical strains could rescue *ntl* expression in epiboly-inhibited embryos. Uniaxial compression resulted in a partial but significant rescue of patterned *ntl* marginal expression, as shown by *in situ* hybridization (Fig. 4c,e and Supplementary Fig. S3f,h) and quantitative reverse-transcription PCR (Fig. 4f and Supplementary Fig. S3i). The re-establishment of the endogenous mechanical strains by drug washing, and magnetic rescue of epiboly of treated embryos, fully rescued *ntl* marginal expression (Figs 3g,h and 4d–f, and Supplementary Fig. S9b). Thus, the onset of epiboly movements induces a specific dilation of marginal cells and provides mechanical signals that trigger β-cat nuclear translocation to initiate *ntl* expression in the presumptive mesoderm of zebrafish embryos.

As above mentioned, such mechanical induction of *ntl* in early mesoderm specification might require additional biochemical signalling, such as Nodal, which is known to be expressed all around the embryo in margin cells at the onset of epiboly^35^,^36^, and to be involved in mesodermal *ntl* expression at the 90% epiboly stage (9hpf)^37^. However, except for the dorsal-most margin, we found no inhibition of *ntl* expression after inhibition of Nodal signalling in the complete mesoderm by treatment with the Nodal receptor inhibitor SB-431542 (ref. 37; Supplementary Fig. S10a,b,g)^38^ and, consistently, no inhibition of β-cat nuclear translocation at the onset of epiboly in most of the margin cells (Supplementary Fig. S10c,d,g). In addition, except at the dorsal pole, the rescue of the β-cat nuclear translocation was still observed in embryos treated with blebbistatin and deformed by uniaxial global compression, in the presence of the Nodal receptor inhibitor SB-431542 (Supplementary Fig. S10e–g). This indicates the requirement of the biochemical factor Nodal for the mechanical induction of β-cat nuclear translocation and *ntl* at the dorsal pole only, but no requirement of Nodal for mechanotransductive β-cat-dependent early-mesoderm *ntl* expression in the majority of margin cells in the ventro-lateral domain.

### Mechanical induction of Twist in *Drosophila* embryo mesoderm

**Figure 5:**
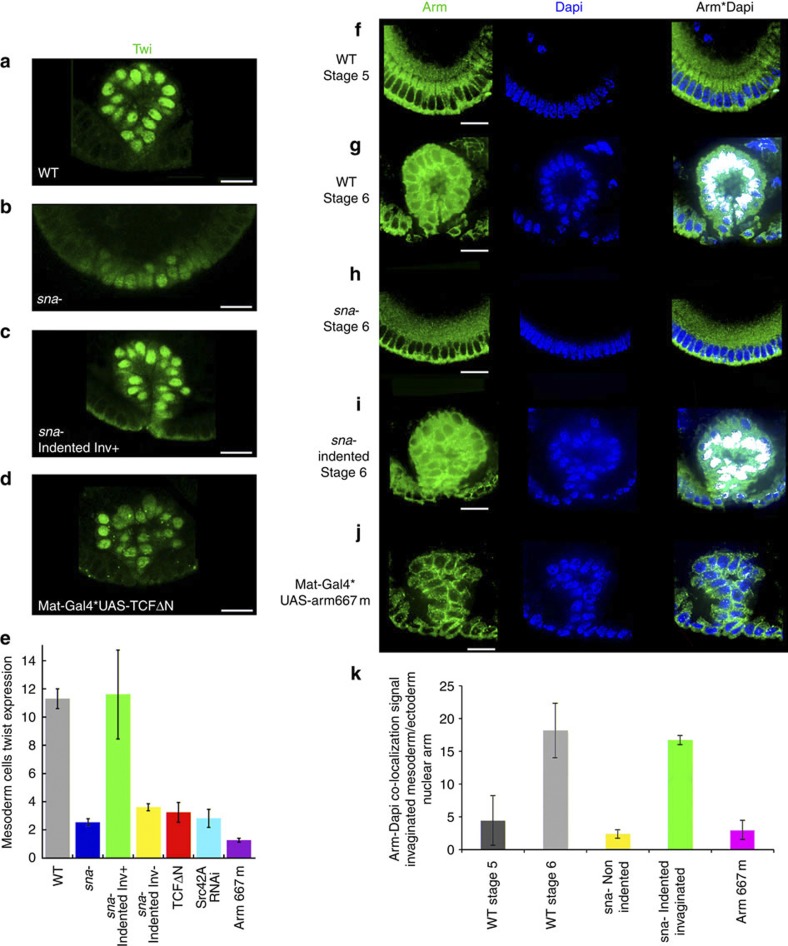
**Mechanical induction of nuclear translocation of β-cat and of Twist expression in the** ***Drosophila*** **embryo mesoderm**. (**a**) Twi expression in the mesoderm of *Drosophila* embryos at stage 8 after WT invagination (*n*=6). (**b**) Twist expression in non-invaginating *sna*^−/−^ mutants (*n*=7). (**c**) Twist expression after invagination rescue through indentation of *sna*^−/−^ mutants (*n*=6). (**d**) Twi expression in the mesoderm of TcfΔN embryos at stage 8 (*n*=6). (**e**) Quantification of Twi expression in the mesoderm of WT embryos (*n*=6), *sna*^*−*^ embryos (*n*=7), *sna*^*−*^ indented embryos with rescue of mesoderm invagination (*n*=6), *sna*^*−*^ indented embryos without rescue of mesoderm invagination (*n*=6), TcfΔN embryos (*n*=6), Src42A-RNAi embryos (*n*=12) and Arm667m embryos (*n*=10). Data are characterized by a Mann–Whitney’s exact test *P*<0.001. Error bars are s.d. (**f**) Arm labelling at stage 5 before mesoderm invagination. (**g**) Arm labelling during mesoderm invagination at stage 6. (**h**) Arm labelling in stage-6 *sna*^*−/−*^ mutants. (**i**) Arm labelling in mesoderm-invaginated *sna* mutants after indentation. (**j**) Arm labelling in Mat-Gal4*Arm667m Y667 unphosphorylable Arm mutants. Note the diffuse texture of the image associated with 1% formaldehyde procedure for which nuclear detection of Arm is possible (see Methods) but junctional resolution is lost. Higher membranar resolution is achieved with the 2% formaldehyde procedure, but characterizes membrane and cytoplasm only (Supplementary Fig. S11d). Note also that in contrast to zebrafish, signalling Arm is known to be more diffusively found in the cytoplasm with some degree of nuclear enrichment only^43^. (**k**) Quantification of Arm nuclear translocation (ratio of average mesodermal co-localization signal (density of white pixels) over ectodermal co-localization signal) in the mesoderm of WT at stage 5 (*n*=7), at stage 6 (*n*=6), in the *sna*^*−*^ at stage 6 (*n*=6), in the *sna*^*−*^ indented with rescue of invagination at stage 6 (*n*=6), in Arm667m at stage 6 (*n*=8). Arbitrary units (a.u.). Data are characterized by a Mann–Whitney’s exact test *P*<0.01 for all relevant comparison. Error bars are s.d. *sna*^*−/−*^ mutants were selected by phenotype *a priori* (no mesoderm constriction and invagination before the onset of germ band extension and a delay of 10 min in anterior gut invagination^14^), as well as *a posteriori* (profound lateral dorsal folds characteristic of *sna* mutants as described in ref. 69) and by Snail labelling (see Supplementary Fig. S11a,b). All embryos are at stage 8. All experiments were replicated twice. Scale bar, 10 μm (white bars).

Mechano-induced β-cat/Armadillo (Arm) nuclear translocation is also known to act in the *Drosophila* anterior midgut to induce the expression of the mesendodermal gene *twist* (*twi*) in response to compression by germ band extension at gastrulation^9^,^13^. In the prospect of making a close comparison with zebrafish, we proceeded to test β-cat/Arm-dependent *twi* mechanosensitivity in the mesoderm of *Drosophila*. The *twi* expression is initiated by the maternal transcription factor Dorsal^39^, but in *snail* (*sna*)-null mutants, which lack mesoderm invagination, *twi* expression vanishes prematurely at stage 8, 45 min to 1 h after WT invagination^40^. This had led to speculation that the mechanical cues associated with mesoderm invagination could act to maintain a high level of *twi* expression in the mesoderm^41^. To test the involvement of a mechanotransduction β-cat/Arm pathway leading to *twi* expression during mesoderm invagination, we rescued the mesoderm invagination in a *sna*^−/−^ mutant, taking advantage of the mechanotransduction response of *sna*^−/−^ embryos to soft indentation. This is known to rescue, with 70% success, the apical accumulation of Myo-II, leading to mesoderm invagination lacking in *sna*^*−/−*^, in a Fog-dependent process^14^. This led to the full rescue of Twi protein levels (Fig. 5a–c,e and Supplementary Fig. S11a–c). To test that β-cat/Arm is involved in the mesodermal Twi mechanical induction process, we quantified Twi expression in Tcf-ΔN-overexpressing individuals. We found that Twist expression dramatically dropped in the mesoderm of transgenic individuals at stage 8, thereby demonstrating the transcriptional activity of Arm in mesodermal Twist expression at gastrulation^42^ (Fig. 5d,e). Immunofluorescence staining confirmed that Arm is released from apical cellular membranes to the cytoplasm (Supplementary Fig. S11d), with translocation observed in the nuclei upon invagination at gastrulation (Fig. 5f,g,k with oil × 60 objective and Supplementary Fig. S12a,b with air × 20 objective, with soft 1% formaldehyde fixation procedure deteriorating junctional labelling resolution but allowing nuclear detection of endogenous Arm). In contrast to strong nuclear labelling in zebrafish, signalling Arm in early *Drosophila* embryos is known to be more diffusively found in the cytoplasm with some degree of localization in the nuclei^43^, consistent with our observations. Arm nuclear translocation was further evidenced by Dapi/Arm co-localization analysis quantification (in white in Fig. 5f–k, see Methods). Arm membrane release from apical junctions to the cytoplasm and nuclei is lost in *sna*^−/−^ mutants and rescued in invaginating indented individuals, demonstrating mechanical induction of Arm release from the junctions to the cytoplasm and nuclei in response to mesoderm invagination strains (Fig. 5h,i,k and Supplementary Figs S11d and S12a,b).

It can be concluded that mesoderm invagination in *Drosophila* provides mechanical signals resulting in Arm junctional release and transcriptional activity, to maintain Twi expression and mesodermal identity after mesoderm invagination.

### Μechanical induction of Twist and *ntl* by p-Y654/667β-cat

In the *Drosophila* anterior midgut, as in mice colon cancer lines, the mechanically induced nuclear translocation of β-cat permissively requires Src family kinases that phosphorylate the tyrosine present in one of the sites of interaction between β-cat and E-cadherin, and represses their interaction^13^,^27^. In mice, the phosphorylation of the β-cat Y654 site by Src family kinases impairs the interaction of β-cat with E-cadherin, leading to the release of β-cat into the cytoplasm and into the nucleus in the case of defective *Adenomatous polyposis coli* (*APC*) degradation^27^. The Arm homologous amino acid of the mouse Tyrosine Y654 is Tyrosine Y667 (ref. 44). Because of the strong conservation of these two phosphorylation sites (Supplementary Fig. S13), we labelled *Drosophila* embryos with the antibody recognizing specifically the phosphorylated form of the mouse Tyrosine Y654 (ref. 27).

We first found mechanical induction of the Src42A-dependent phosphorylation of the β-cat tyrosine 667 (pY667-β-cat) interaction site in the early *Drosophila* embryo (Supplementary Fig. S14a–g). Effectively, we found high labelling in mechanically compressed *Drosophila* embryos compared with non-compressed embryos (Supplementary Fig. S14a,b,g). Such labelling vanished in compressed Mat-Gal4*UAS-Arm667m embryos overexpressing an Arm form in which Y667 is substituted by a non-phosphorylable phenylalanine (Supplementary Fig. S14c,d,g), demonstrating that the p-Y654 anti-mouse antibody recognizes the *Drosophila* pY667 site, and that the Y667 site is phosphorylated in response to mechanical strains in *Drosophila* embryos. In addition, we found that overexpressing Src42A-RNAi in Mat-Gal4*UAS-SrcRNAi represses the mechanical phosphorylation of the Y667 site (Supplementary Fig. S14e–g). Src42A is thus required for the mechanical phosphorylation of the Y667 site observed in the WT. In addition, as for Twist mechanical induction in the future anterior midgut stomodeal cells^13^, Src42A is already phosphorylated (activated) at stage 5 before gastrulation and is not overactivated by the morphogenetic movement of mesoderm invagination (Supplementary Fig. S15a). Src42A is thus not activated by mechanical strains but is only permissive in the mechanotransduction process.

**Figure 6:**
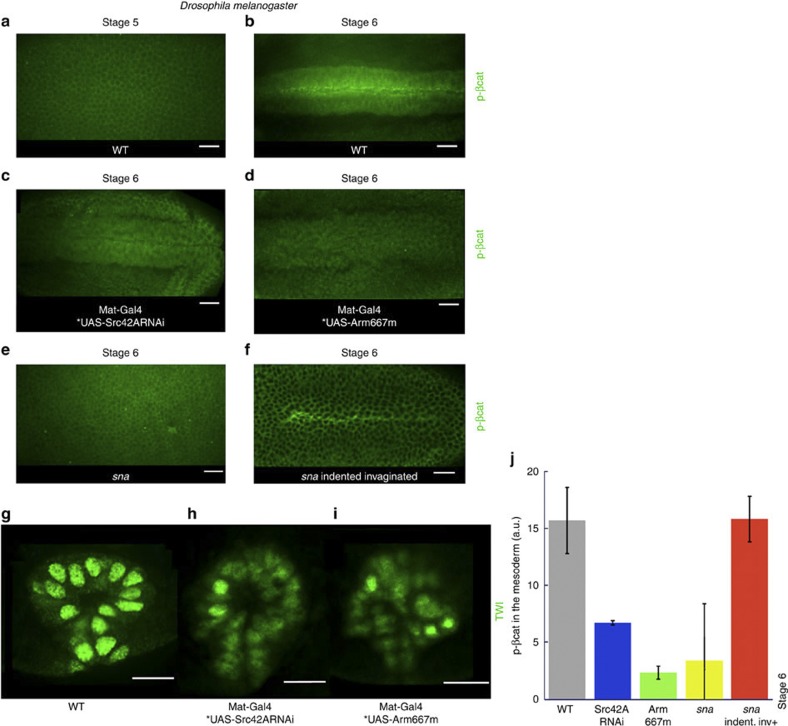
**Mechanically induced phosphorylation of the Y667-β-cat E-cadherin interaction site in the mesoderm of** ***Drosophila***. (**a**) Phospho-Y667-β-cat labelling in stage-5 *Drosophila* embryos. (**b**) Phospho-β-cat labelling in stage-6 invaginating embryos. (**c**) Phospho-β-cat labelling in Src42A-RNAi invaginating embryos. (**d**) Phospho-β-cat labelling in Arm667m stage-6 invaginating embryos. (**e**) Phospho-β-cat labelling in the non-invaginating mesoderm of stage 6 *sna*^*−/−*^ *Drosophila* embryos. (**f**) Phospho-β-cat labelling in the indented invaginating *sna*^−/−^ mutants rescued in mesoderm invagination (note mesoderm invagination rescue can also be shallower than WT, see Supplementary Fig. S11b). Scale bar, 20 μm (white bars). (**g**) Twist expression in the WT at stage 8. (**h**) Twist expression in Src42A-RNAi at stage 8. (**i**) Twist expression in Arm667m embryos at stage 8. See Fig. 5e for quantification. Scale bar, 10 μm (white bars). (**j**) Quantification of Arm Y667 phosphorylation in the ventral furrow of *Drosophila* WT embryos (*n*=18), in Src42A-RNAi (*n*=13), in Arm667m (*n*=8), in *sna*^−/−^ embryos (*n*=7) and in indented invaginated *sna*^−/−^ embryos (*n*=6). *P*<0.001 according to Mann–Whitney’s exact test. Error bars are s.d. All experiments were replicated two times.

**Figure 7:**
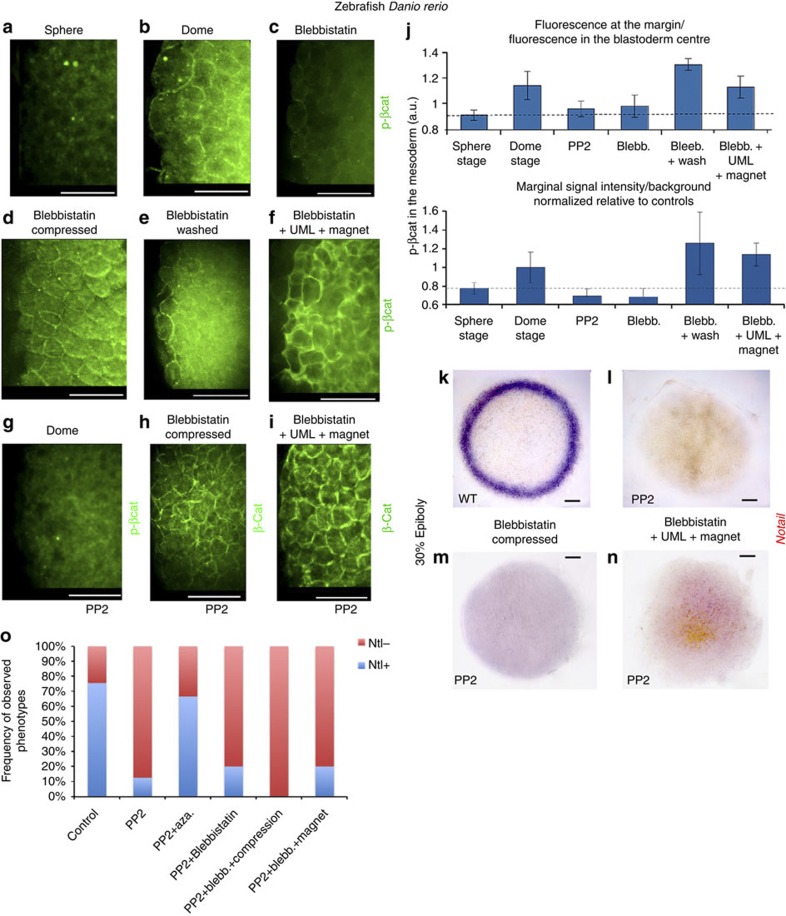
**Mechanically induced phosphorylation of the Y667-β-cat conserved E-cadherin interaction site in the mesoderm of** ***Danio***. (**a**) Phospho-β-cat labelling in zebrafish marginal cells before epiboly (sphere stage). (**b**) Phospho-β-cat labelling at the start of epiboly (dome stage). (**c**) Phospho-β-cat labelling after blebbistatin treatment that suppressed movements. (**d**) Phospho-β-cat labelling after blebbistatin treatment with epiboly movements rescued by global compression. (**e**) Phospho-β-cat labelling after blebbistatin treatment with epiboly movements rescued by drug washing. (**f**) Phospho-β-cat labelling after blebbistatin treatment rescued by magnetic manipulation of UML-injected embryos leading to epiboly movement resumption. (**g**) Phospho-β-cat labelling in the presence of PP2 Src-family inhibitor treatment at dome (associated β-cat nuclear translocation tests in Supplementary Fig. S15b). (**h**) β-cat labelling in blebbistatin globally compressed embryos treated with PP2. (**i**) β-cat labelling in blebbistatin magnetically deformed UML-injected embryos treated with PP2. Associated quantitative results in Supplementary Fig. S15b. (**j**) Levels of pY667 β-cat in the margin relative to the blastoderm centre and in the margin relative to the background in sphere stage (*n*=6), dome stage (*n*=9), PP2 treated (*n*=7), blebbistatin-treated (*n*=22), blebbistatin-treated and washed (*n*=7), and blebbistatin-treated UML-injected magnetically rescued embryos (*n*=9). Differences between control dome, blebbistatin-washed or blebbistatin-compressed embryos and all other conditions are statistically significant (*P*<0.05 according to Mann–Whitney’s exact test. Error bars are s.d. All experiments were replicated two times. (**k**) *ntl* expression at 30% epiboly. (**l**) *ntl* expression at 30% epiboly upon PP2 treatment in blebbistatin-treated embryos. (**m**) *ntl* expression at 30% epiboly upon PP2 treatment in blebbistatin-treated embryos with margin cell epiboly deformation rescued by global compression. (**n**) *ntl* expression at 30% epiboly upon PP2 treatment in blebbistatin-treated embryos with margin cell epiboly deformation rescued by magnetic forces. (**o**) Proportion of embryos showing *ntl* expression in controls (*n*=49), PP2-treated embryos (*n*=8), PP2- and 1-azakenpaullone-treated embryos (*n*=9), PP2- and blebbistatin-treated embryos (*n*=15), PP2 and blebbistatin globally compressed embryos (*n*=18), PP2 and blebbistatin UML-injected embryos submitted to the ring magnet (*n*=5). All data are characterized by *P*<0.05 by *χ*^2^-test. All experiments were replicated two times. Scale bar, 20 μm (white bars) and 100 μm (black bars),

We then found that immunostainings for Y667 phospho-β-cat yield a strong localized signal in the mesoderm during invagination at stage 6 in a Src42A- and Y667-dependent way (Fig. 6a–d,j). Consistent with this, apical release of Arm from membranes to the nuclei and late Twist expression are lost in a Src42A- and Y667-dependent way (Figs 5e,j,k and 6g–i, and Supplementary Figs S11d and S15a). Furthermore, Arm Y667 phosphorylation was absent in *sna*^*−/−*^ mutants and restored on invagination rescue after soft indentation (Fig. 6e,f,j). In zebrafish, a statistically significant 20% increase in the conserved phospho-Y654 β-cat concentration can be detected by immunofluorescence at the marginal zone during the first 5 min of epiboly, predominantly in junctions (Fig. 7a,b,j). It is suppressed by blocking epiboly and is rescued by compression, washing and magnetic manipulation (Fig. 7c–f,j). Note that the phospho-Y654 β-cat is more concentrated in the cortex, suggesting a process of rapid dephosphorylation after release from the membrane. Consistently with *Drosophila*, β-cat phosphorylation, nuclear translocation and *ntl* expression at 30% epiboly are abolished by PP2 Src family kinase inhibitor treatment at the onset of epiboly (Fig. 7g,k,l,o, Supplementary Figs S15b and S16a–h). Neither could be re-established in blebbistatin-treated embryos rescued with global compression or magnetic forces in the presence of PP2 (Fig. 7h–j,m–o and Supplementary Fig. S15b), showing the requirement of Y654 β-cat phosphorylation for β-cat-dependent *ntl* mechanical induction.

**Figure 8:**
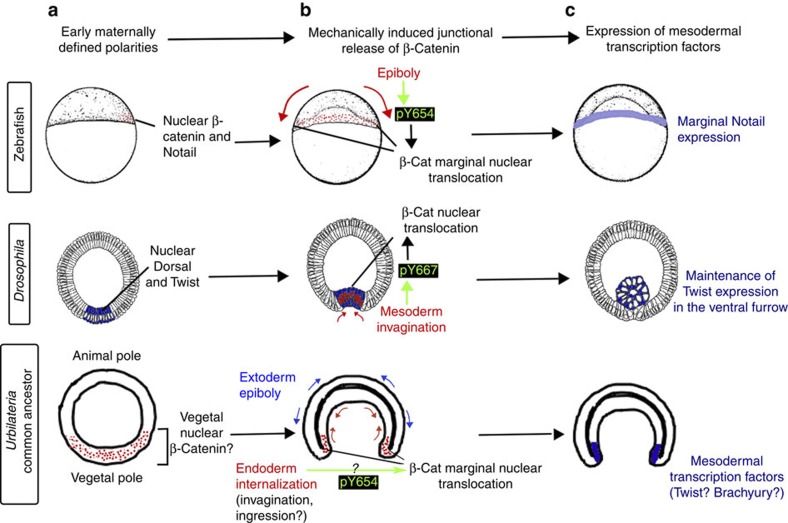
**Gastrulation induced pY667β-cat leads to early mesoderm specification in zebrafish and** ***Drosophila*** **that have diverged at the time of mesoderm emergence**. (**a**) In both *Danio* and *Drosophila*, early maternally defined polarities allow the first morphogenetic movements of gatrulation that lead to specific deformation of mesodermal cells. (**b**) This deformation causes the mechanically induced Src-family-kinase-mediated phosphorylation of Y667-β-cat, its junctional release, nuclear translocation and transcriptional activity. (**c**) This results into either induction (*ntl* in zebrafish) or maintenance (*twi* in *Drosophila*) of early panmesodermal transcription factors. These strikingly parallel situations probably date back to Urbilateria, the last common ancestor of bilaterians, in which the mesoderm has been proposed to have arisen from the margin of the balstopore, which is by defiinition a strongly deformed zone.

These results demonstrate that in both *drosophila* and zebrafish, β-cat phosphorylation is mechanically induced in a Src-dependent process. This allows β-cat to be released from junctions to the nucleus and leads to the stimulation of mesoderm transcription-factor expression during gastrulation (Fig. 8a–c).

Here we find that the triggering of the zebrafish *ntl* expression and the maintenance of the *Drosophila* Twist expression in presumptive mesoderm cells are mechanically induced in a β-cat dependent mechanotransductive process by the first morphogenetic movements of gastrulation in both species. We demonstrate that the phosphorylation of the Y667 site of β-cat that prevents the interaction with E-cadherin is the key mechanotransduction molecular event, leading to the release of β-cat from the junctions to the cytosol leading to nuclear translocation and expression of mesodermal genes in both species. We conclude that an identical mechanosensitive pathway acts in early mesoderm development in two distantly related bilaterians, *Drosophila* and *Danio.* This detailed similarity indicates common inheritance of this pathway from the Urbilateria, the last common Protostomia–Deuterostomia bilaterian ancestor.

## Discussion

The search for a common biochemical pathway leading to mesoderm formation across Bilateria has so far proved to be difficult^1^,^15^,^21^,^34^, especially owing to the case of the protostome superphylum Ecdysozoa, for which there has been no reports of a role for β-cat in early mesoderm specification^16^. As we demonstrate Y667/654-β-cat phosphorylation-dependent mechanical induction of early mesoderm specification to be a common pathway in two such distantly related species as *Drosophila* and zebrafish, thereby introducing a role for β-cat signalling in early mesoderm specification in a member of Ecydosozoa—that is, *Drosophila*—we suggest that mesoderm specification by mechanical signals could be of ancient bilaterian origin.

If the mechanotransduction pathway we describe dates back to the Urbilateria, the last common ancestor of Protostomes (*Drosophila*) and Deuterostomes (zebrafish), there should be potential for its conservation in other Bilateria. Indeed, our results have interesting parallels in other organisms. In *Xenopus*, it was shown that the marginal nuclear translocation of β-cat at stage 9 was surprisingly independent of Wnt ligands and necessary for mesoderm induction^21^. Strikingly, this coincides temporally with the first detectable morphogenetic movements in the *Xenopus* ectoderm^45^. In artificially bent *Xenopus* animal explants, mesodermal identity is mechanically induced in the convex (dilated) side^46^. In sea urchins, *bra* is downstream of the Wnt canonical β-cat pathway and is strictly expressed at the highly curved deformed margin of the blastopore with extinction of transcription on internalization, an observation that could be elegantly explained by control through mechanical signals^47^. In amniotes, such as mice and chicken, the movements of epiboly (that happen at the rim of the blastoderm) are uncoupled from the process of mesoderm induction which, instead, takes place at the primitive streak^20^. Interestingly, the primitive streak is established by strong morphogenetic movements (known as polonaise in chick embryos), which might provide the required strains for mechanical mesoderm induction in amniotes. More investigations are required to test this point. So far, data obtained in mouse indeed confirm that the transcription activity of β-cat is required for the initiation of *bra* expression^22^. These observations suggest broad conservation for the mechanotransduction pathway we describe at least among deuterostomes.

From a morphogenetic point of view, gastrulation movements are strikingly diverse across Metazoans, including invagination, epiboly-driven involution, ingression and delamination. Is this diversity compatible with the idea of a conserved requirement for mechanical cues in the establishment or maintenance of gene expression patterns? In this respect, we note that the two bilaterians we compare, *Drosophila* and zebrafish, display strikingly divergent modes of gastrulation, as *Drosophila* gastrulate only by invagination while the zebrafish embryo is strictly epibolic at our stage of interest. Indeed, *Drosophila* and zebrafish are practically as different, as far as modes of gastrulation are concerned, as two bilaterians can possibly be. Nevertheless, these radically different morphogenetic movements are compatible with conservation of the same mechanosensitive pathway during mesoderm specification. This indicates that the conservation of mechanotransduction pathways, even though it constantly requires cell deformation, is not tightly constrained by the exact type of morphogenetic movement involved. In the case we report here, it is sufficient that mesoderm cells undergo a specific and localized deformation before or during mesoderm specification. The comparison between the two species we studied demonstrates that this is compatible with important divergences in morphogenetic mechanisms.

From a mechanistic point of view, the phosphorylation of the Y654 β-cat site was found in different contexts to reduce β-cat affinity with E-cadherins and in several cases to be sufficient to trigger nuclear translocation^48^,^49^,^50^,^51^. Within the present mechanotransductive context, how cytoplasmic β-cat, having been released from the junctions after Y654 phosphorylation, escapes Axin/APC-mediated degradation to translocate in the nucleus in the mesoderm is an interesting question that will be addressed in future investigations. It might either be due to a parallel mechanical inhibition of the APC/Axin complex, a process known in other biochemical contexts^52^, or to an overflow of cytoplasmic β-cat due to a burst of mechanical signals that could saturate and bypass the degradation machinery and lead to partial nuclear translocation, as observed in Fig. 5g,i. Consistently with the latter hypothesis, it is worth noting that in the case of *Drosophila*, weakly observable β-cat nuclear translocation^43^ is known to be sufficient for transcriptional activation, with β-cat transcriptional activity having been demonstrated by defects in target gene expression in TCF dominant-negative mutants^42^,^53^ (Fig. 5d,e).

From an evolutionary perspective, our results are consistent with the reconstituted ancestral fate map of bilaterians, where the mesoderm is proposed to originate at the blastoporal margin, which, indeed, is by definition a strongly deformed zone (Fig. 8)^54^. Adding to the cell–cell adhesion ancient metazoan role of β-cat allowing the constitution of epithelia^55^, we thus propose that in the last common ancestor of protostomes and deuterostomes, mechanical deformation associated with gastrulation at the blastoporal margin could have induced Y667/654 phosphorylation of β-cat and nuclear translocation, and, in turn, activated the expression of mesodermal transcription factors. In conjunction with the mechanical induction of β-cat transcriptional activity, additional signalling pathways such as Nodal^37^ and Dorsal^16^ were differentially recruited to contribute to mesoderm induction in independent branches of the animal evolutionary tree, probably consolidating the output of the ancestral mechanotransduction pathway. Indeed, Nodal and Dorsal have no conserved role in mesoderm determination in *Drosophila* and zebrafish, respectively^56^,^57^. In contrast, here we find that mechanical induction of Y667/654 phosphorylation of β-cat in early mesoderm determination is conserved in both species. This suggests that the mechanotransductive β-cat pathway is more ancient than the protostome-specific or deuterostome-specific pathways previously identified in mesoderm induction, and dates back to the last common ancestor of zebrafish and *Drosophila* more than 570 million years ago, the period during which the mesoderm is thought to have emerged^58^,^59^. A conserved mechanotransductive origin of the mesoderm specification in Bilateria embryos thus suggests that the mesoderm could have developed in diploblastic ancestors through the emergence of the mechanical control of the phosphorylation of the β-cat Y667/654 site in response to gastrulation morphogenetic movements.

## Methods

### Zebrafish epiboly inhibition

For our experiments, we did not use larvae that have reached an ‘independent feeding’ stage. Therefore, according to the DIRECTIVE 2010/63/EU and its implementation in France, no prior authorization for this project was required and this study was not submitted to our respective Ethical Committee (National registration number: 118) for ethical approval. Nevertheless, the Animal Welfare Body of the Institut Curie (IC) Research Center regularly checked that all the husbandry practices meet the ethical requirements. Epiboly was inhibited by treating chorionated zebrafish embryos at the early sphere stage (3.8 hpf) with 50 μM blebbistatin or 66 μM (20 μg ml^−1^) nocodazole dissolved in 5 ml embryo medium. Nocodazole and blebbistatin were purchased from Sigma Aldrich, dissolved in dimethylsulphoxide (DMSO) to a final concentration of 5 (blebbistatin) or 10 (nocodazole) mg ml^−1^ and stored at −20 °C as single-use aliquots. Both drugs completely inhibited epiboly up until 3 h after the start of control epiboly, a stage at which treated individuals develop a small epiboly that does not proceed further than 30%. Blebbistatin treatment was performed 15 min at midsphere before mechanical treatment, in the dark as much as possible to avoid drug photodestruction. During compression assays, a 620-nm high-pass filter was used to prevent ultraviolet light from degrading blebbistatin. Drug washing was performed by removing the embryo medium and rinsing the eggs five times with embryo medium. Movements were rescued in 100% of blebbistatin-treated batches after 5 min. Nocodazole washing did not rescue epiboly before 2 h and was thus not tested. Control embryos were systematically treated with an equivalent quantity of DMSO (10–14.6 μl in 5 ml embryo medium), which yielded no observable developmental defect.

### Zebrafish Wnt pathway inhibition

β-cat-mediated transcription was inhibited in zebrafish embryos by the use of a heat-shock-inducible dominant-negative Tcf–GFP (HS-TcfΔN-GFP) construct^23^ (lent by Nicolas David, ENS Paris). Wnt receptors were inhibited by a heat-shock-inducible Dkk-GFP fusion protein (HS:Dkk-GFP; courtesy of Gilbert Weidinger, Technische Universität Dresden). WT females were crossed with heterozygous transgenic males and the resulting progeny was heat shocked for 1 h at 38 °C starting at the sphere stage. Resulting embryos were sorted out according to GFP fluorescence to separate transgenic individuals from the WT siblings used as controls. GFP-positive and GFP-negative individuals both identically performed epiboly after heat shock. *Dkk1* mRNA injections (respectively HS:Dkk-GFP induction assays) were given according to Hashimoto *et al.*^60^ and were validated *a posteriori* by observation of the described *dkk1* overexpression phenotype at 24 hpf in at least six or seven individuals left to develop from each injected (respectively, heat shocked) and immunostained batch. In the case of the HS:Dkk-GFP construct, the immunostaining protocol (see Supplementary Methods ‘Zebrafish embryo fixation and immunostaining’) was modified in the following way to allow *a posteriori* imaging and quantification of the GFP signal: the methanol storage step was skipped to avoid methanol-induced degradation of GFP, the staining protocol was entirely performed in the dark to prevent photobleaching and a Cy3-anti-mouse secondary antibody (Jackson) was used for β-cat detection to allow simultaneous imaging of β-cat and Dkk-GFP.

Note that chemical inhibitors of the Wnt pathway (XAV939 for transcription inhibition and IWP2 for Wnt secretion inhibition) purchased from Sigma Aldrich were tested on dechorionated zebrafish embryos from sphere stage onwards at the maximal concentrations achievable, with stock solutions prepared according to the manufacturer’s instruction (amounting to a final concentration of 1% DMSO) without yielding any phenotype in 24-hpf-treated embryos, indicating that these chemical inhibitors are impractical for Wnt inhibition at this stage and at these concentrations on zebrafish embryos, and that transgenic strains are required for this purpose.

### Zebrafish Wnt pathway activation

The Wnt pathway was activated in zebrafish by chemical inhibitors of GSK-3β. LiCl (0.3 M) treatments were performed as described by Stachel *et al.*^32^, for 10 min. LiCl treatments were started on epiboly-blocked embryos when control siblings had reached the dome stage, and embryos were fixed at various stages after treatment. Embryos were fixed 1 or 2 h after the start of the treatment. Results could be seen at both fixation times but were stronger after 2 h (all phenotypes shown were obtained after 2 h). Alsterpaullone and 1-azakenpaullone were purchased from Sigma Aldrich, dissolved in DMSO into 10 mM stock solutions and stored at −20 °C. As they were found to have no effect on chorionated embryos, embryos were systematically dechorionated before treatment with both activators. Alsterpaullone (1 μM) treatments were pursued for 1 h, which killed nearly 50% of the treated embryos. Higher concentrations or longer treatment times resulted in killing nearly all individuals. 1-Azakenpaullone (10 μM) treatments in combination with nocodazole were pursued for 2 h before fixation, with little toxicity. 1-Azakenpaullone (1 μM) in combination with blebbistatin was toxic to all individuals. In all figures, epiboly-inhibited and Wnt-rescued embryos were fixed at the same time. Control embryos were systematically treated with an equivalent quantity of DMSO, which yielded no observable developmental defects.

### Zebrafish Src-kinase inhibition

Src family kinases were inhibited by the chemical inhibitor PP2 purchased from Sigma Aldrich. PP2 was dissolved in DMSO into 83 mM stock solutions that were stored at −20 °C. Embryos were dechorionated before treatment and treated with 200 μM PP2 from the sphere stage onwards. PP2 treatment did not detectably affect epiboly, thus ruling out nonspecific toxic effects. Control embryos were systematically treated with an equivalent quantity of DMSO, which yielded no observable developmental defects.

### Zebrafish Nodal pathway inhibition

To inhibit activity of the Nodal pathway, zebrafish embryos were dechorionated and treated from dome stage onwards with 800 μM of SB-431542 purchased from Sigma Aldrich (stored as a 100 mM stock in DMSO at −20 °C), following a published protocol^37^.

### Global compression mechanical deformation of zebrafish

To rescue cellular deformations in epibolyless embryos, 35 μm z-deformation uniaxial global compression of chorionated embryos were first realized on an agar plate with a coverslip handled by a micromanipulator^9^. Compression was systematically started in samples showing no epiboly initiation after drug treatments, simultaneously with the initiation of epiboly (dome stage) in non-treated control siblings. Embryos were photographed before and during compression (see Supplementary Fig. S17a,b, respectively). Compression was performed for 5 min before p-Y654-β-cat for 20 min followed by a 10-min relaxation time before β-cat staining, and for 1 h followed by 30 min of relaxation for the assessment of *ntl* expression. The global compression induced a dome shape change characteristic of the initiation of epiboly movement. PIV analysis of the border cells observed in immunofluorescence (up to the dashed line of Fig. 2c) showed a border cell physiological deformation of 4% min^−1^ as described in the principal text (in this experiment, cells immediately below the dashed line partially disappear out of focus inside the embryo, leading to non-meaningful compression patterns in red). Embryos were fixed immediately at the end of the relaxation phase.

### Magnetic deformation of zebrafish

A radially magnetized ring micromagnet of average cross section 50 μm and inner diameter 800 μm was prepared using the recently developed micro-Magnetic Imprinting technique^61^. The overall fabrication process contains two distinct phases, namely template preparation (steps 1 and 2) and polymer composite preparation (steps 3 and 4). In the first step, high-rate triode sputtering was used to deposit an out-of-plane textured hard magnetic NdFeB film^62^. In the second step, the film was magnetized in the out-of-plane direction (+z), and then a stripe of width 20 μm and length 2.5 mm was re-magnetized in the opposite direction (−z) using the Thermo-Magnetic-Patterning technique^63^. In this technique, pulsed ultraviolet–laser irradiation is performed through a micropatterned mask to locally heat and, thus, reduce the coercivity of the film to allow magnetization reversal in an external magnetic field that is weaker than the film’s room temperature value of coercivity. In the third step, commercially available coercive particles of NdFeB of average size 15 μm ( http://www.mqitechnology.com) were sprinkled onto the film surface and were trapped along the re-magnetized stripe, as the magnetic field and magnetic-field gradient is maximum at the interface between oppositely magnetized regions^64^. In a fourth step, the magnetic particles are enrobed in a PDMS (polydimethylsiloxane) matrix, the overall thickness of which was reduced to 100 μm by spin coating. Once the PDMS matrix was cured, the composite PDMS/powder sheet was peeled off the hard magnetic film that served as a template and the embedded particles were magnetized in the direction perpendicular to the sheet plane. In a fifth and final step, the sheet was rolled up to produce a cylinder of inner diameter 800 μm, the spatially organized magnetic particles serving to form a radially magnetized ring magnet and the PDMS cylindrical matrix serving as a transparent, flexible support for the ring magnet.

Ten nanolitres of a 1.5-M solution of fluorescent UMLs^65^ was injected at the one-cell stage and the embryo was left to develop until blebbistatin treatment at the early sphere stage (Supplementary Fig. S18a). The embryo was then placed inside the PDMS cylinder containing the embedded ring micromagnet so that the axis of symmetry of the magnet is concentric with the embryo (Supplementary Fig. S18a,b). Simulations indicate that the stray field produced by the ring micromagnet at the position of the margin cells, that is, 400 μm above the ring, is characterized by a relatively low B.gradB value of 10^−2^ T^2^ m^−1^. This led to a slow variation of the mean local deformation of the blastoderm border (0.25±0.06 μm min^−1^ (*n*=4)), quantitatively mimicking the movements measured at the onset of epiboly at dome formation (0.22±0.04 μm min^−1^ (*n*=5); Fig. 3c,d). No detectable movement was observed in the blebbistatin-treated embryos. Interestingly, even the most pronounced mutant of epiboly (Poky) is characterized by fluctuating border cell movements of an amplitude of the order of 1 μm% min^−1^ (ref. 66), showing the necessity of the use of drug treatment to address the question of mechanical induction at the onset of epiboly. In addition, the use of a macroscopic magnet, which gave a B.gradB value of 1 T^2^ m^−1^ resulted in movements of margin cells leading to tissue destruction (not shown), demonstrating the necessity to use the microscaled ring to quantitatively reproduce the soft epiboly movements in drug-treated embryos, *in vivo*. No movement is observed in blebbistatin embryos after UML injection without exposure to the micromagnet, nor with exposure to a magnet without injection (not shown). Note that the curved PDMS matrix containing the ring micromagnet degraded the optical resolution such that PIV could technically be not realized in this configuration. Note that when injected in one cell at a four-cell stage, the injected volume was 2.5 nl.

### Zebrafish deformation analysis

Zebrafish embryos were stained with 2 μM FM-464 (Molecular Probes) and imaged using an Olympus IX70-Ropper spinning-disk microscope coupled to a Cool Snap HQ2 camera on excitation by a 405-nm laser after embedding in 1% low-melting-point agarose. The cells imaged were systematically located in the marginal zone and in the plane immediately below the enveloping layer, in deep cells belonging to presumptive mesendoderm. Resulting pictures were processed by MatPIV with coarse graining (a PIV software package written by Johan Kristian Sveen for use with Matlab)^13^. Deformation patterns were obtained by calculating the mean value of the velocity field divergence over 4 min. Compression (positive deformation) is red and dilation (negative deformation) is blue. The red line indicates the limit between the marginal cells and the yolk syncitial nuclei.

### Fluorescence quantification

Fluorescent signals were quantified using the software ImageJ and were systematically normalized with respect to the extracellular background. For the zebrafish measurements that were restricted to the marginal zone, the margin was defined as the zone that displays β-cat nuclear translocation in controls from the same batch, which corresponds to the three to four most marginal rows of cells from 4.3 to 4.7 hpf.

In the specific case of mechanical rescue of β-cat nuclear translocation in blebbistatin-treated embryos (Fig. 2), the experiment was realized in a double-blind manner, with experiments realized at the IC by AB, labelling of the samples realized at the University Pierre et Marie Curie by CY and sent back to the IC under encoded names, with imaging and analysis of the samples realized in blind at the IC by AB. Conditions of experimentation of the labelled embryos (CY) and results (AB) were finally compared collectively (CY and AB) at the end of the blind procedure.

Sample sizes depended on the maximal number of embryos that could be treated simultaneously (6 for magnetic deformation and about 15 for global compression) and was validated *a posteriori* by statistical significance of the effects measured. The variance was similar within any group of data. Significance of the data was assessed using the Mann–Whitney exact test, and was double checked with Student’s *t*-test after assessing normality of the data set using Shapiro–Wilk’s normality test for each data series (*e*-value>1). In any case, error bars represent s.d.

### Fly strains

Oregon-R (used for WT) and SnaIIG/Cyo *sna* mutants were from Bloomington. Stocks were maintained at room temperature (typically 22 °C) and experiments were carried out at 25 °C, except for UAS*Gal4 crosses, which was stored 2 h at 28 °C before experiments. UAS-dTCFΔN expresses a 31-amino-acid amino-terminal truncation of dTCF and was purchased from Bloomington. UAS-Arm667m was donated by Pernille Rorth^44^. Mat-Gal4 (III) (matα4-GALVP16/V37 tubulin) was provided by Bloomington. Homozygous UAS-Src42A/RNAi were produced by crossing 7873R-2 Src42A/RNAi with UAS-Src42A-RNAi (II) provided by the NIG Stock Centre and VDRC Stock Centre, respectively.

### *Drosophila* immunostaining

Embryos were dechorionated and fixed for 30 min at the interface of heptane/4% formaldehyde PIPES, and at heptane/2% formaldehyde PIPES for membrane and cytoplasmic Arm labelling (to be as close as possible to the 1% formaldehyde conditions allowing nuclear Arm detection with formaldehyde alone^9^,^13^, and still allow transverse mechanical cutting). Antibody staining was done in PBT (PBS, 0.2% Tween and 1% BSA). The heptane/1% formaldehyde PIPES conditions allowing Arm nuclear detection were used for the first time, to our knowledge, for mesoderm observations, with 4% formaldehyde post fixation after labelling, allowing mechanical cutting of the embryos before observation—except for the Mat-Gal4*UAS-Src42A-RNAi conditions that did not resist mechanical cutting. This fixation procedure is in contrast to the heat MeOH rapid fixation known to extract most of the cytosolic Arm so as to strongly enhance junction labelling compared with cytosolic labelling^67^ (especially using peroxidase amplification). The Arm-GFP could not be used, being non-functional for nuclear translocation in *Drosophila* embryos (personal communication, D. McEwen and M. Peifer). Proteins were detected with the appropriate antibodies: rabbit anti-Twist, donated by Siegfried Roth (Köln University, Germany, dilution 1:5,000); guinea pig anti-snail, donated by Yutaka Nibu (Cornell University, USA, dilution 1:200); mouse anti-phospho-Y654-β-cat, Abcam (dilution 1:50); rabbit anti-phospho-dSrc42A, donated by Shigeo Hayashi (dil 1:125) and mouse anti-Arm antibody, DSHB (dilution 1:200). Twist and p-Src42A were detected using Alexa 488 anti-rabbit secondary antibodies purchased from Molecular probes (dilution 1:100); Snail antibody was detected with Cy3-anti-guinea pig from Molecular probes; P-Y654-β-cat antibody was detected using Cy3-anti-mouse antibodies purchased from Jackson (dilution 1/500); Arm antibody was detected by anti-mouse-fluorescein isothiocyanate secondary antibodies from Vector (dilution 1:50). Embryos were mounted in Vectashield (Vector Laboratories). Mechanical cross-cuts were realized, embryo by embryo, using a scalpel, and mounted vertically in the Vectashield for confocal observation. Images were obtained with an inverted Olympus IX70-Ropper spinning-disc microscope coupled to a Cool Snap HQ2 camera. Images were processed with Metamorph or Adobe Photoshop software.

### *Drosophila* mechanical deformation

*snail^*−/−*^ Indents:* Rescue of invagination in *sna* mutant embryos was performed by indentation^14^. Early stage-5 dechorionated embryos were glued laterally on a coverslip recovered of dried heptane scotch. A micromanipulated 50-μm-diameter needle was approached close to the mesoderm. Embryos having not initiated invagination 3 min after the end of ventral cellularization were genotyped as *sna*^*−/−*^*sna*^*−*^ and were systematically indented by 3–7 μm during 4 min at the most posterior 1/3 position of the mesoderm. Basically, in the WT, *sna* expression leads to active fluctuations of mesoderm cells that are thought to activate the Fog mechanosensitive pathway that leads to apical stabilization of Myo-II and mesoderm invagination. This proposal was based on experiments demonstrating the Fog-dependent rescue of apical stabilization of Myo-II and mesoderm invagination lacking in *sna* mutants, by indenting the *sna* mutant mesoderm^14^.

*snail^*−/−*^ Phenotypes criteria:* As Snail expression fades rapidly after mesoderm invagination^68^, the criteria to select *sna*^*−/−*^ homozygous were phenotypic *a priori* (no mesoderm constriction, no invagination at germ band-extension initiation and delay of 10 min in the formation of the anterior midgut, following Pouille *et al.*^14^) and *a posteriori* (formation of two anomalous profound lateral folds at stage 8 (ref. 69), see Supplementary Fig. S11a), in addition to immunofluorescent labelling of Snail.

### Fluorescence quantification

Regarding Twist expression quantification, fluorescent signals were measured using Imaris software. Automatic recognition of nuclei shape allowed systematic measurement of all mesoderm positively labelled nuclei (I+) and all lateral ectoderm negatively labelled control nuclei (I−), from three-dimensional spinning-disc imaging of fixed embryos labelled with a-Twi. The signal was calculated following I+/I−. The Imaris analysis and calculations were automatic and realized in blind by BD. Note that no decrease of Twist expression was observed in Mat-Gal4 controls alone at 28 °C compared with WT at 25 °C, only a small increase of 13%, probably due to a temperature increase. In the specific case of *sna* mutant indented without mesoderm invagination rescue that showed partial but significant rescue of Twist expression (Fig. 5e, yellow and Supplementary Fig. S11a,c), the experiment was realized in a double-blinded manner, with experiments realized at IC by PA, labelling realized at Institut Jacques Monod by AP in a blinded manner and automatic analysis by Imaris realized at Institut Jacques Monod and IC in a double-blinded manner by BD (see Supplementary Fig. S11c). Conditions of experimentation (PA) and results (BD and AP) were finally compared together (PA, BD and AP) at the end of the blind procedure.

Regarding junctional phospho-βcat analysis (Fig. 6j), the mesoderm signal was subtracted from the ectoderm signal and were normalized to the external background. In globally compressed embryos in which all the tissue responded homogeneously throughout the tissue, and more importantly in the junctions (Supplementary Fig. S14), the junctional signal was subtracted from the cytoplasmic signal for optimization of the normalization of individual embryo-labelling fluctuations, and normalized to the external background, with the result of the compressed embryos normalized to the uncompressed control. In the latter specific case, error bars were s.e.

Quantification of nuclear Arm translocation was performed as follows: images of Arm-immunostained and 4',6-diamidino-2-phenylindole (DAPI)-stained cross-sectioned *Drosophila* embryos were acquired at oil × 60 magnification using an Olympus spinning-disc microscope. Nuclear Arm translocation was evidenced using the Co-localization Highlighter function of the Co-localization Analysis ImageJ plugin ( http://www.uhnresearch.ca/facilities/wcif/imagej/colour_analysis.htm). The sensitivity settings of the Co-localization Highlighter were adjusted as follows: the threshold for the DAPI channel was set at the minimal value that retains only the nuclear signal over extranuclear background and the threshold for the Arm channel was set at the minimal level that leads to the observation of a weak white background around the nuclei of ectodermal cells. Co-localization was coded in a binary way as white pixels (positive for co-localization) and quantified by measuring the density of white pixels over the mesodermal nuclei, normalized by the density of white pixels over the ectodermal nuclei.

At air × 20 magnification, DAPI hardly delimits the nuclei; hence, the shape of the nuclei was directly observed and selected for specific measurements of nuclei signal after systematic screening of the signal in *z*. In that case, the nuclear signal of mesodermal cells was subtracted from yolk noise and divided by the negative nuclear signal of ectodermal cells subtracted from yolk noise.

Note that in contrast to zebrafish, signalling Arm in early *Drosophila* embryos is known to be cytoplasmic with some detectable nuclear translocation^43^.

Sample sizes depended on the maximal number of embryos that could be treated and analysed at the same time and was validated *a posteriori* by statistical significance of the effects measured. The variance was similar within any group of data. In Fig. 5e *sna−* indent inv+, a higher error bar interestingly shows the additional fluctuations introduced after external mechanical manipulation, with statistical significance of the results according to a non-parametric statistic exact test. The significance of all data was assessed using the Mann–Whitney’s exact test, and was double checked with the Student’s *t*-test after assessing normality of the data set using Shapiro–Wilk’s normality test for each data series (*e*-value>1). Error bars represent s.d. All experiments were replicated independently at least two times with similar results in all cases. No sample was excluded from all analysis.

## Author contributions

T.B. and A.B. realized most of the zebrafish experiments, with F.S., I.R. and L.H. having initiated the PIV and the β-cat/nocodazole experiments, respectively, with validation in blind realized by C.Y. G.B. and C.M. produced the UMLs. D.L.-R., F.D.-B., D.G. and N.M.D. designed and developed the ring micromagnets used to produce magnetic forces in the zebrafish experiments. A.B. realized the magnetic rescue experiments. P.A. realized the *Drosophila*/Twi experiment, and B.D. with D.M. realized the *Drosophila*/β-cat experiments, with validation in blind realized by A.P., who also participated in the initial setting up of the fluorescence quantification. T.B. and E.F. designed the zebrafish experiments, interpreted the results in an evolutionary framework and wrote the manuscript. E.F. designed and coordinated the overall zebrafish versus *Drosophila* research.

## Additional information

**How to cite this article:** Brunet, T. & Bouclet, A. *et al.* Evolutionary conservation of early mesoderm specification by mechanotransduction in Bilateria. *Nat. Commun.* 4:2821 doi: 10.1038/ncomms3821 (2013).

## Supplementary Material

Supplementary Information Supplementary Figures S1-S18, Supplementary Methods and Supplementary References
