# Supplementary material for: Evolutionary conservation of early mesoderm specification by mechanotransduction in Bilateria
Source: Nat Commun. 2013 Nov 27;4:2821. doi: 10.1038/ncomms3821 (PMC3868206; doi:10.1038/ncomms3821)
Supplement: Supplementary Information — Supplementary Figures S1-S18, Supplementary Methods and Supplementary References [file ncomms3821-s1.pdf]

Supplementary Figures:

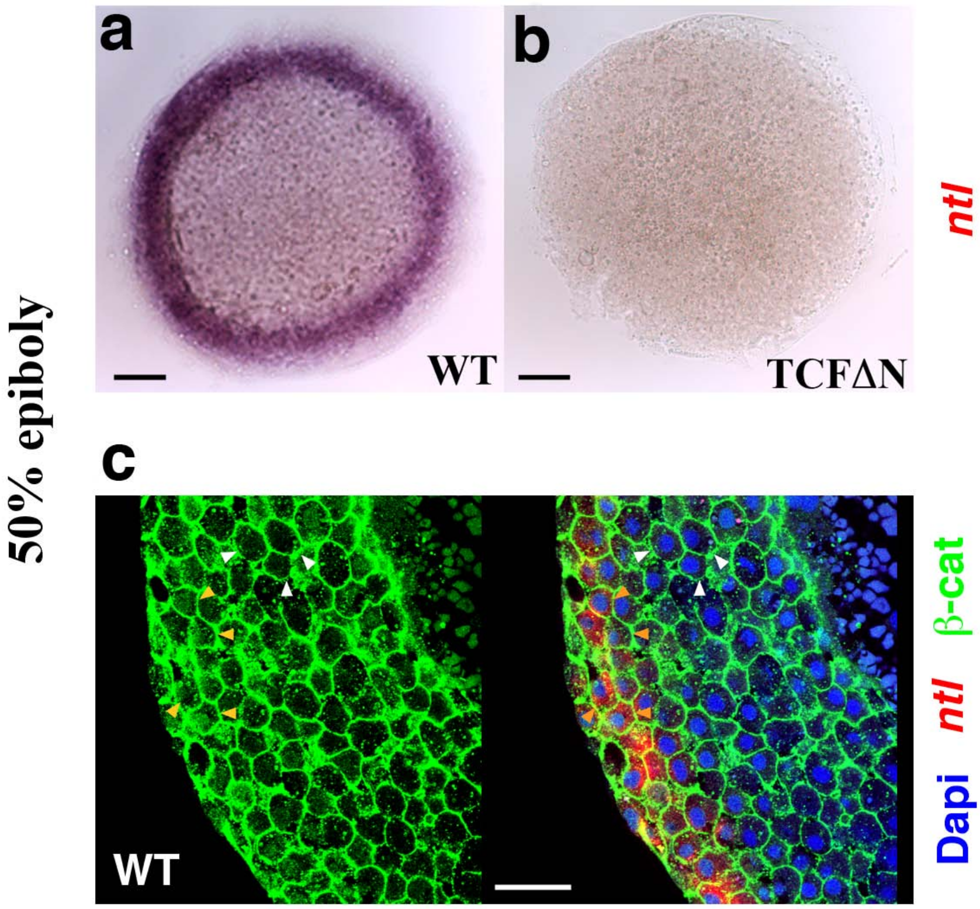

Supplementary Fig. S1

***ntl* expression at the marginal zone of 50% epiboly zebrafish embryo requires β-cat transcriptional activity** (**a**) *ntl* is detectable by *in situ* hybridization on heat-shocked wild-type siblings at 50% epiboly (79/79). (**b**) *ntl* is undetectable by in situ hybridization on heat-shocked HS-TcfΔN-GFP transgenic embryos (26/26). Experiments were replicated 2 times. (**c**) Confocal image of the marginal cells of a wild-type embryo (dome stage) labelled for β-catenin (green), *notail* in situ hybridization signal (red) and nuclei (DAPI, blue) (5/5). Note that the *notail* signal is only detected in cells displaying nuclear β-catenin (orange arrowheads), while cells without detectable nuclear β-catenin (white arrowheads) are devoid of *notail* signal. This suggests that β-catenin is necessary for *notail* expression in a cell-autonomous way. Some β-catenin-positive cells seem *notail*-negative, which might reflect the inability of the in situ hybridization procedure to detect low *notail* expression levels, or indicate that nuclear β-catenin alone is not sufficient to turn on *notail* expression and requires additional cofactors. Note that the *ntl in situ* labelling procedure is not optimal for β-catenin labelling and that β-catenin labelling is consequently less bright than in single labelling conditions. The black bars are 100 microns and the white bar is 20 microns.

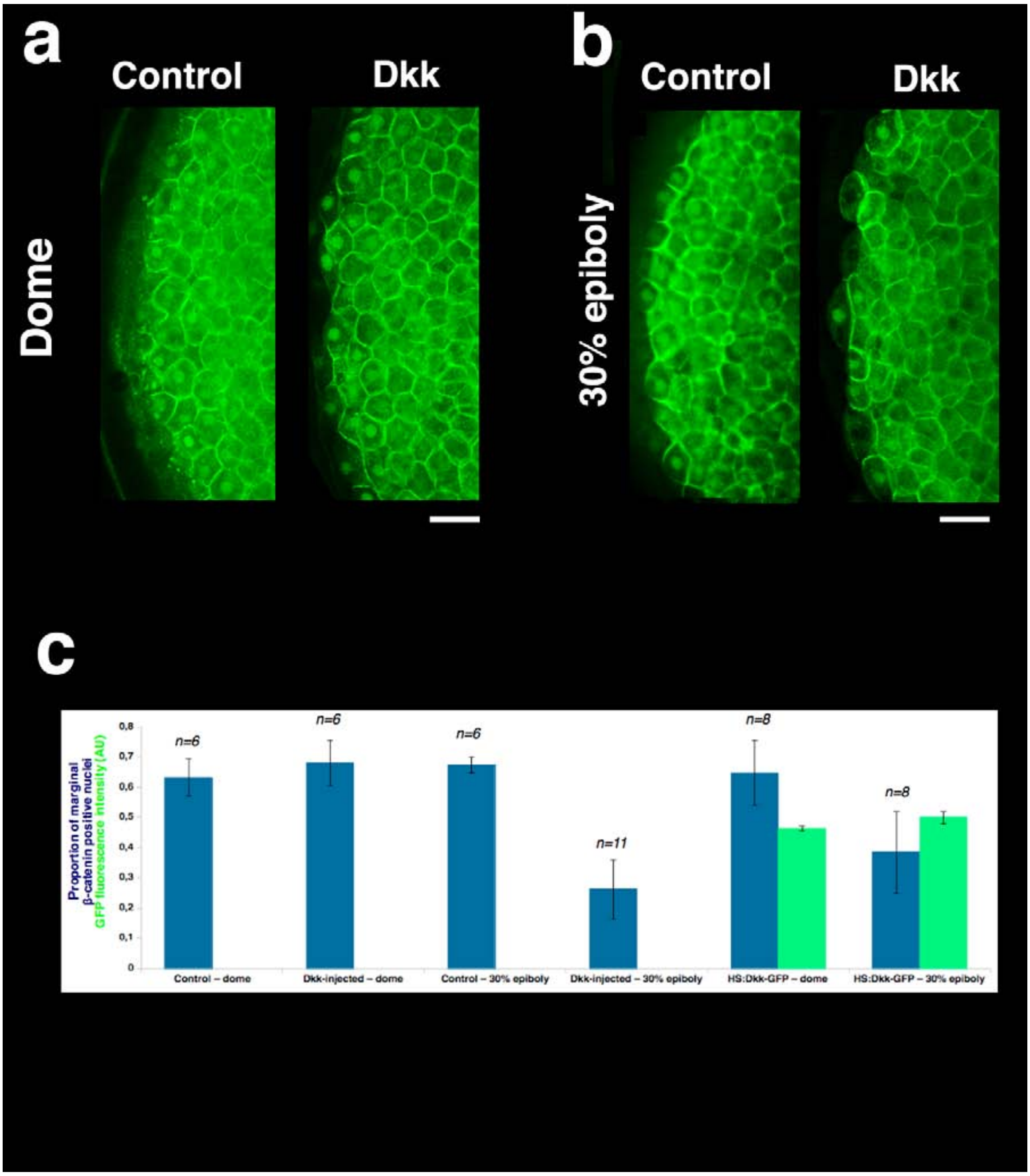

Supplementary Fig. S2

Wnt ligands are not required to establish, but only to maintain, marginal nuclear translocation of β-cat during zebrafish epiboly (**a**) Marginal nuclear translocation of β-cat is clearly visible in control dome stage zebrafish embryos. Marginal nuclear translocation of β-cat is not affected at dome stage by inhibition of Wnt co-receptors by *dkk* injection or *dkk-GFP* heat shock induced expression. (**b**) Marginal nuclear translocation of β-cat persists at 30% epiboly in control embryos. Marginal nuclear translocation of β-cat is severely reduced at 30% epiboly in *dkk*-injected embryos. (**c**) Quantification of marginal nuclear translocation of β-cat under the different conditions studied. Mann-Whitney's exact test *p*-values are 0.29 at dome stage and $1.04*10^{-5}$ at 30% epiboly (comparing controls and *dkk*-injected embryos) and 0.43 at dome stage and $2.4*10^{-2}$ at 30% epiboly (comparing controls and HS:Dkk-GFP transgenic embryos). All experiments were replicated 2 times. Error bars are standard deviation. The white bar is 20 microns.

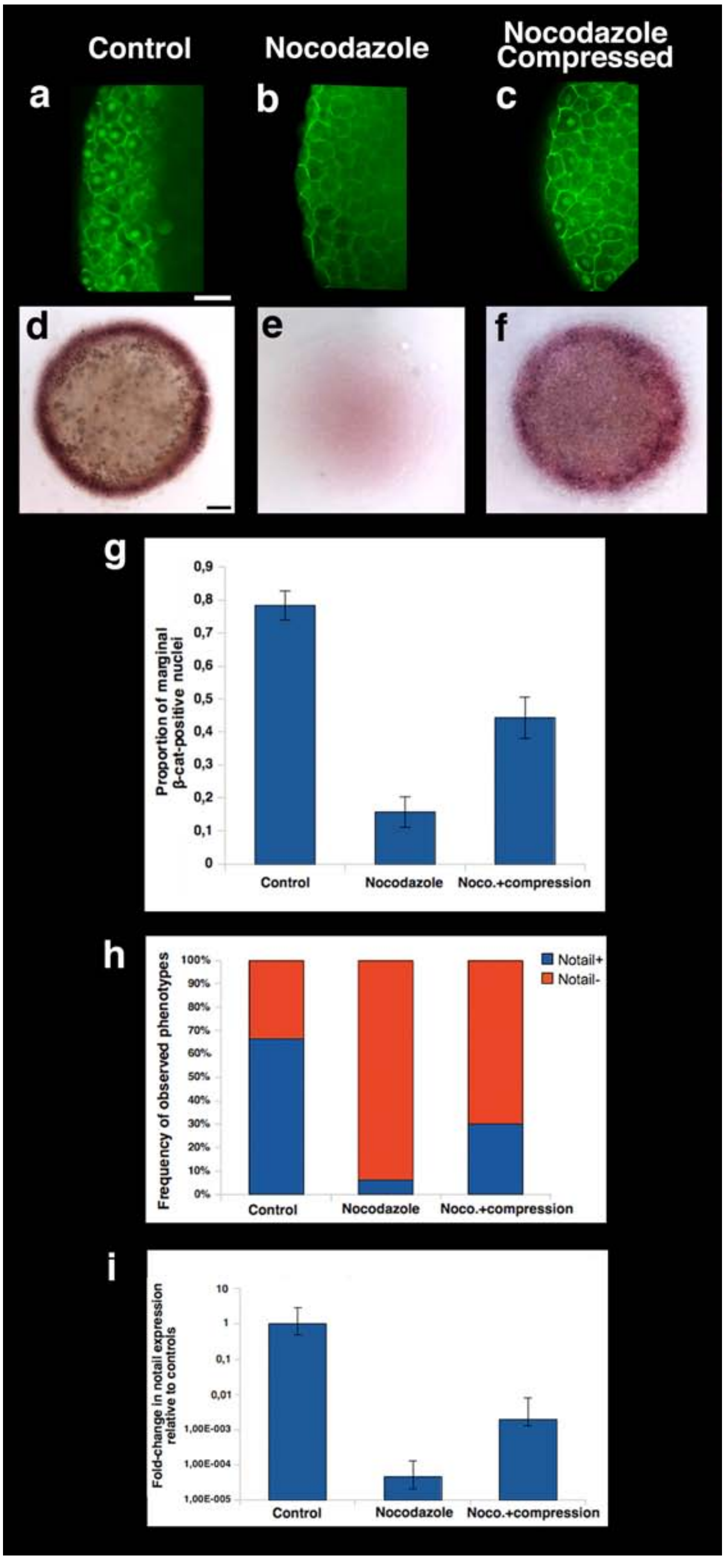

Supplementary Fig. S3

Nocodazole-mediated epiboly inhibition provides independent confirmation of the blebbistatin results (**a**) Marginal nuclear translocation of β-cat in 30% epiboly control embryos. (**b**) Marginal nuclear translocation of β-cat is abolished in nocodazole-treated embryos when control siblings reach 30% epiboly. (**c**) Global compression rescues nuclear translocation of β-cat in nocodazole-treated embryos. (**d**) Marginal *ntl* expression in 50% epiboly control embryos. (**e**) *ntl* expression is similarly abolished by nocodazole when control siblings reach 50% epiboly. (**f**) Global compression rescues *ntl* marginal expression in nocodazole-treated embryos. (**g**) Quantification of the number of β-cat positive nuclei in the control (n=5), nocodazole treated (n=5) and nocodazole treated compressed embryos (n=6). Differences between control and blebbistatin or nocodazole-treated embryos, and between treated and rescued embryos, are statistically significant according to Mann-Whitney's exact test ($p<0.001$). Error bars are standard error. All experiments were replicated 2 times. Note that ectopic positive nuclei in blebbistatin-treated and compressed individuals (Fig. S5g) are not taken into account in this quantification. (**h**) Quantification of the number of embryos expressing *ntl* in the control (n=90), nocodazole treated (n=96) and nocodazole treated compressed embryos (n=30). $p<0.05$ by the χ^2-test. All experiments were replicated 2 times. (**i**) RT-qPCR quantification of *ntl* expression in controls, nocodazole treated, nocodazole treated compressed embryos (every reaction realized in technical triplicates). All experiments were replicated 2 times. The white bar is 20 microns and the black bar is 100 microns.

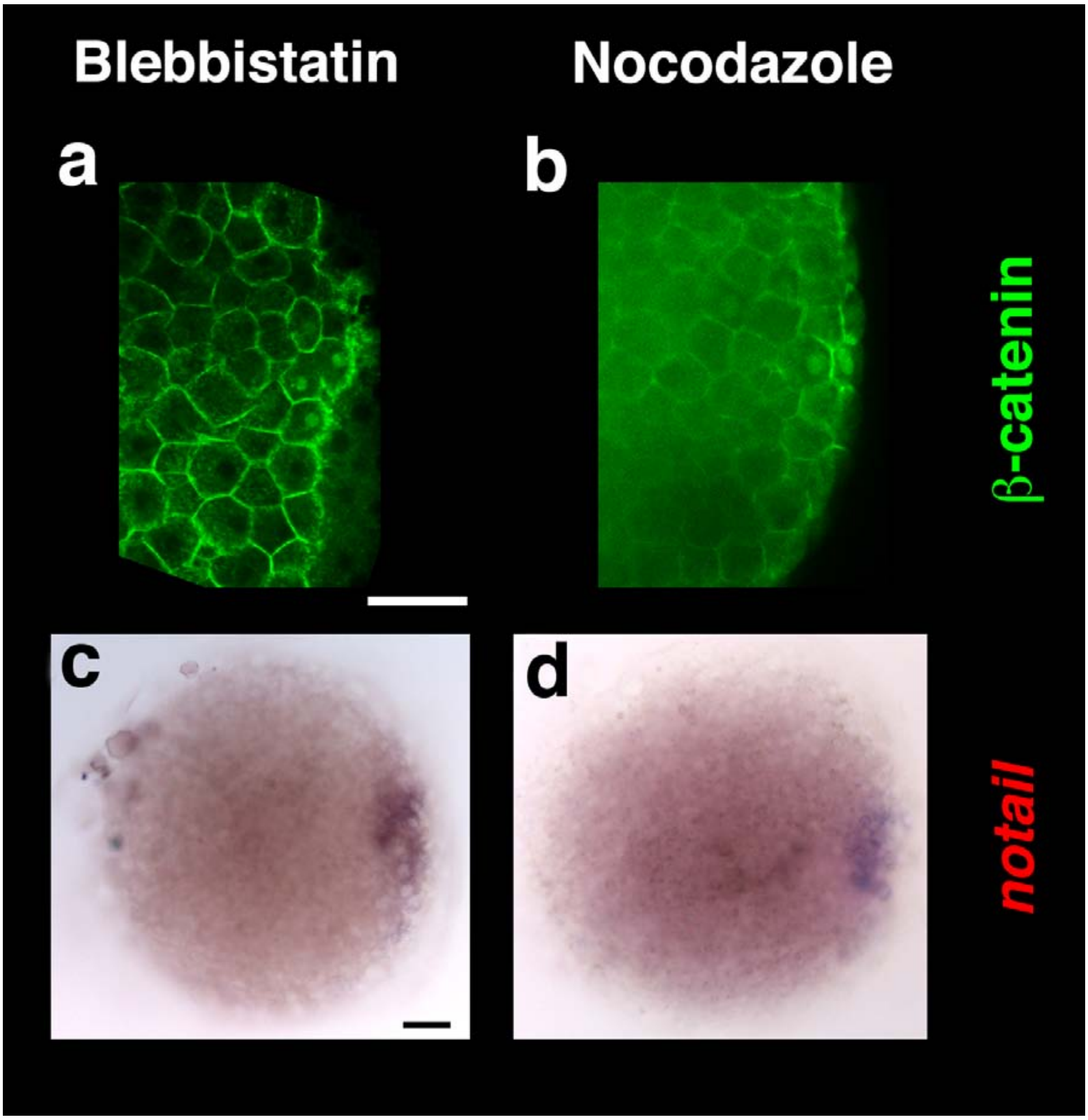

Supplementary Fig. S4

Nuclear translocation of β-cat and gene expression in the dorsal organizer are not affected by epiboly inhibition (**a**) Dorsal nuclear translocation of β-cat is not affected by blebbistatin treatment (5/5) (**b**) Dorsal nuclear translocation of β-cat is not affected by nocodazole treatment (8/8). (**c**) When control siblings reach dome stage, blebbistatin-treated embryos display persistent unipolar, presumably dorsal, expression of *ntl* (8/10). (**d**) An identical phenotype is observed in nocodazole-treated embryos at the same stage (9/12). The nocodazole treatment often led to a weak additional noise compared to blebbistatin treatment. Note that even the most severe mutant of epiboly, *poky*, showed important local fluctuating movements at the margin[67], preventing the use of mutants to block any movement at epiboly (see M for quantification of *poky* movements). All experiments were replicated 2 times. The white bar is 20 microns and the black bar is 100 microns.

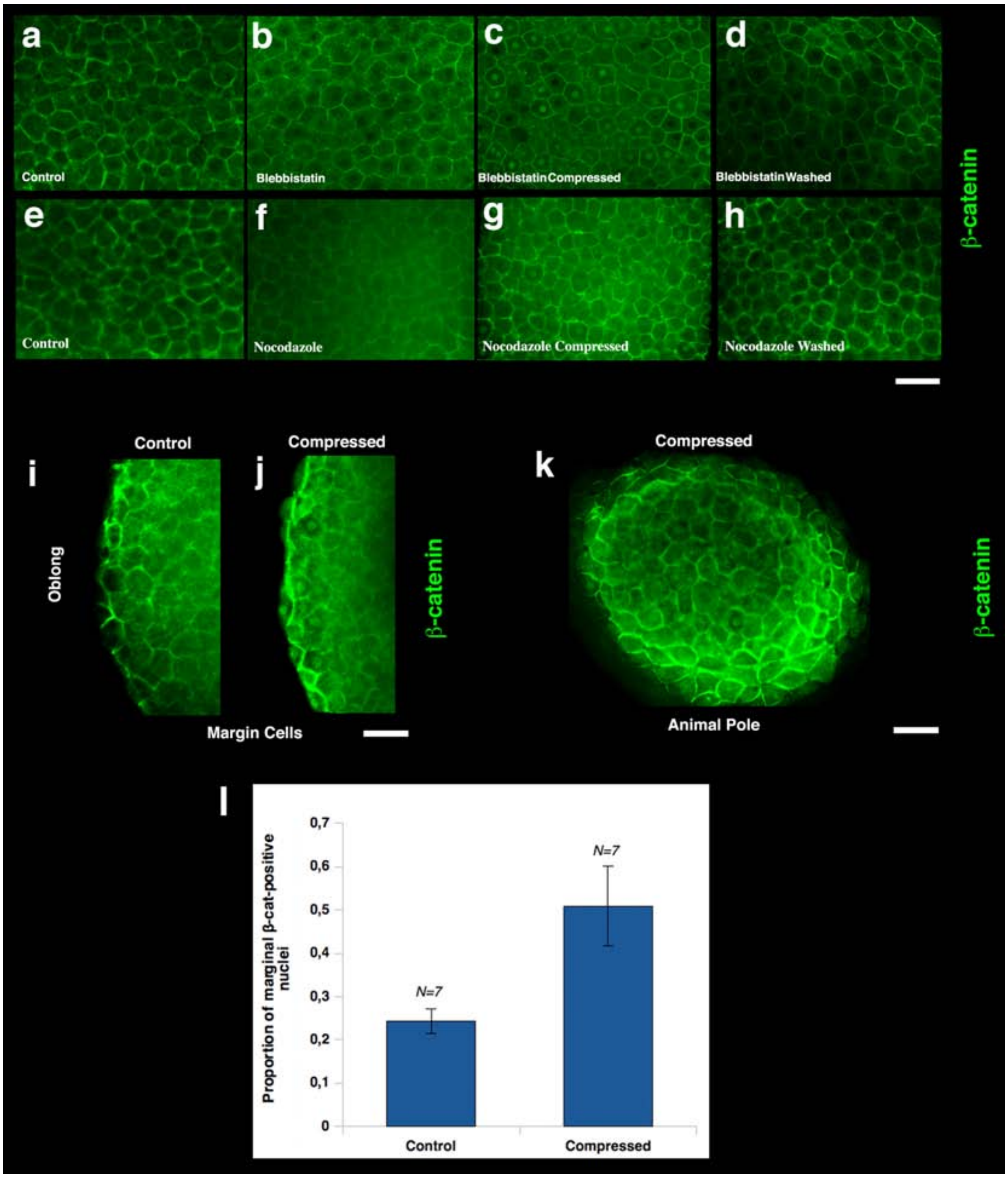

Supplementary Fig. S5

Global compression results in some ectopic nuclear translocation of β-cat at the animal pole in zebrafish embryos (**a**) In control and (**b**) blebbistatin-treated, (**c**) not in blebbistatin-treated compressed individuals in which some nuclear β-cat translocation can be detected at the animal pole, (**d**) and in blebbistatin-treated and washed embryos, no ectopic β-cat-positive nuclei can be detected at the animal pole. (**e**) In control and (**f**) nocodazole-treated, (**g**) not in nocodazole-treated compressed individuals in which some nuclear β-cat translocation can be detected at the animal pole, (**h**) and in nocodazole-treated and washed embryos, no ectopic β-cat-positive nuclei can be detected at the animal pole. Note that nocodazole treatment often led to a weak additional noise compared to blebbistatin treatment. (**i**) No nuclear translocation of β-cat is observed at the margin out of the dorsal pole before the initiation of epiboly of dome stage (shown here at oblong stage) (n=20). (**j**) Rescue of margin nuclear translocation of β-cat in oblong stage compressed embryos. (**k**) Slight nuclear translocation is observed at the animal pole (n=24). (**l**) Quantification of nuclear translocation of β-cat induced by compression in pre-epiboly embryos (N=7) compared to control (n=7). Results are statistically significant according to Mann-Whitney's exact test ($p<0.004$). Error bars are standard deviation. All experiments were replicated 2 times. The white bar is 20 microns.

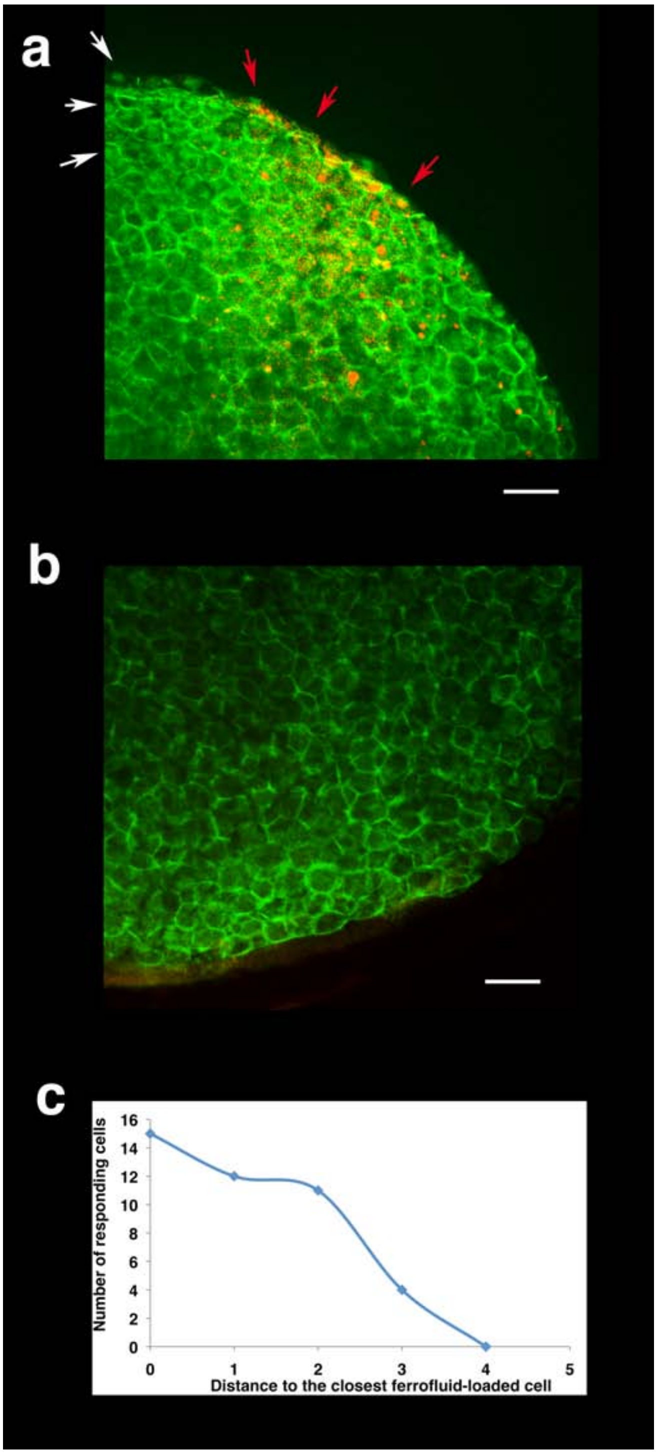

Supplementary Figure S6.

Local injections in one cell of 4-cells-stage embryos reveal range of action of non-cell-autonomous mechanical cues β-catenin immunostaining (green) of an epiboly defective dome stage embryo treated with blebistatin locally injected with fluorescent magnetoliposomes (red) at the 4-cells stage, and submitted to the magnetic field gradient at equivalent dome stage. (**a**) Few-cell-deep vegetal view in the magnetically loaded domain. Note β-catenin nuclear translocation in magnetically loaded cells (red arrows), but also non-magnetically loaded cells (white arrows) as far away as 3 cells distant from the loaded cells. (**b**) No β-catenin nuclear translocation is observed more far away from the magnetically loaded domain. (**c**) Quantification of the action range of non-cell-autonomous mechanical cues. The white bars are 20 microns. Representative of n=6 embryos on the N=6 embryos injected.

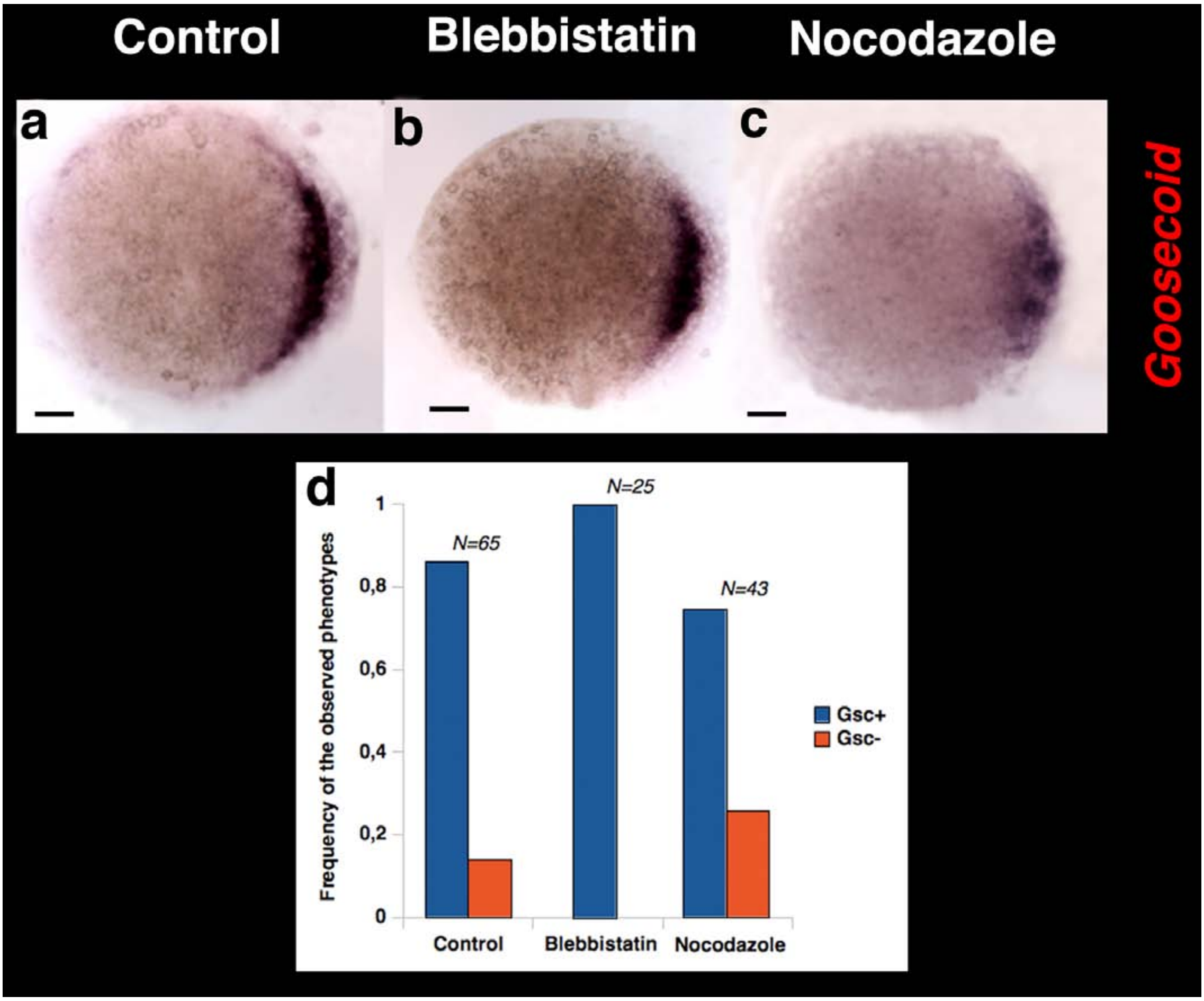

Supplementary Fig. S7

Dorsal *gsc* expression is not abolished by epiboly inhibition in treated embryos (**a**) *gsc* expression at 50% epiboly in control embryos. (**b**) *gsc* expression appears unaffected in both pattern and intensity in blebbistatin-treated embryos. (**c**) *gsc* expression appears unaffected in pattern, but with detectably lower intensity, in nocodazole-treated embryos. (**d**) Frequencies and statistical significance of the observed phenotypes. p=0.19 (resp. 0.11) for the control/nocodazole (resp. control/blebbistatin) comparison, indicating no statistically significant difference between the samples. All experiments were replicated 2 times. The black bar is 100 microns.

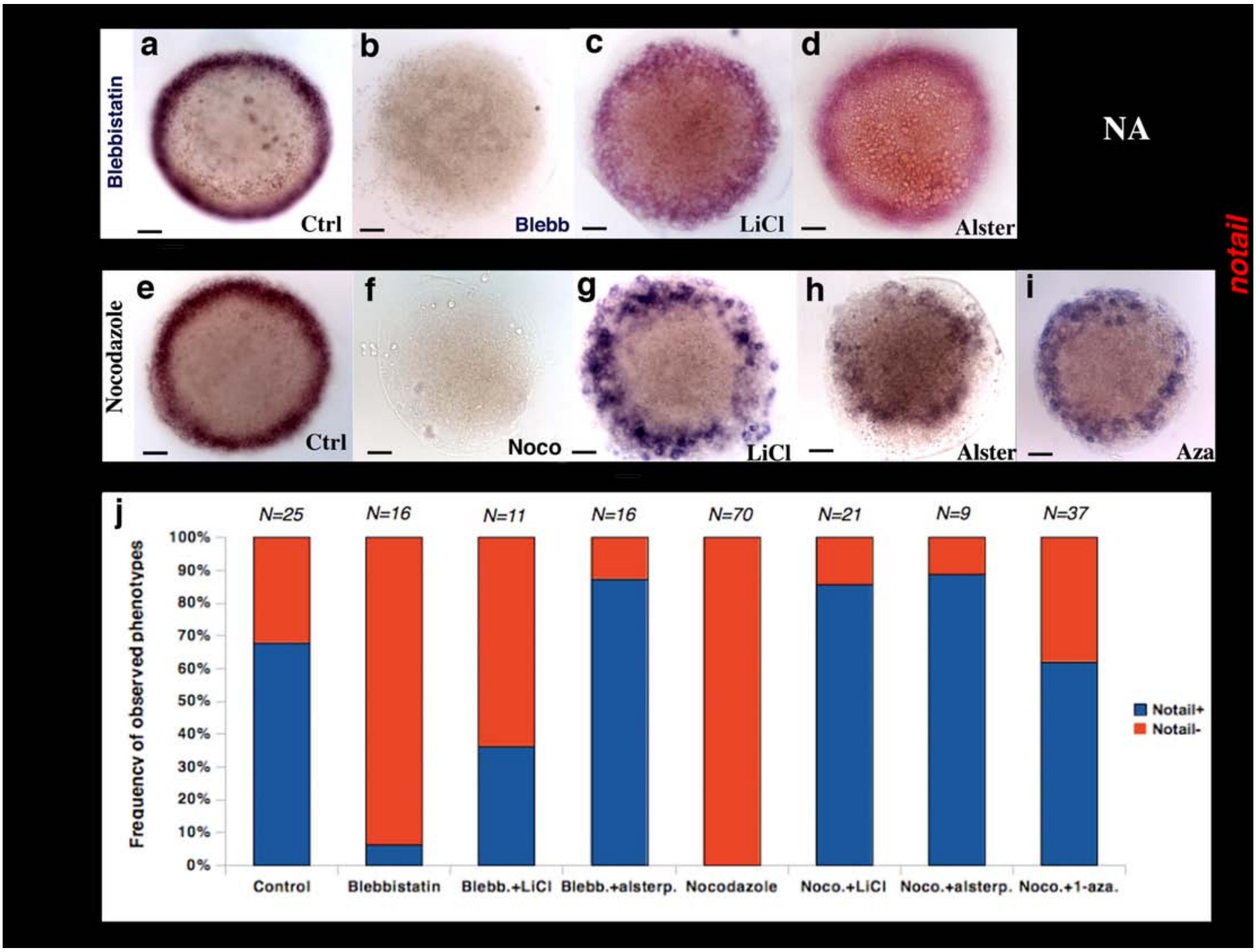

Supplementary Fig. S8

Marginal *ntl* expression is rescued in epiboly-inhibited embryos upon chemical GSK3β inhibition

(a) Marginal expression of *ntl* (**b**) is abolished upon epiboly inhibition by blebbistatin treatment, and can be rescued by chemical stimulation of β-cat nuclear translocation by GSK3β inhibitors: (**c**) LiCl and (**d**) alsterpaullone. (**e**) Marginal expression of *ntl* (**f**) is abolished upon epiboly inhibition by nocodazole treatment, and can be rescued by chemical stimulation of β-cat nuclear translocation by GSK3β inhibitors: (**g**) LiCl, (**h**) alsterpaullone (**i**) and 1-azakenpaullone. The combination of 1-azakenpaullone and blebbistatin turned out to be toxic at the concentrations used. (**j**) Frequencies and statistical significance of the phenotypes observed. χ^2 tests gave p<0.0001 for comparisons between control embryos and epiboly-inhibited embryos, as well as between epiboly-inhibited embryos and epiboly-inhibited embryos treated with GSK3β inhibitors. All experiments were replicated 2 times. The black bars are 100 microns.

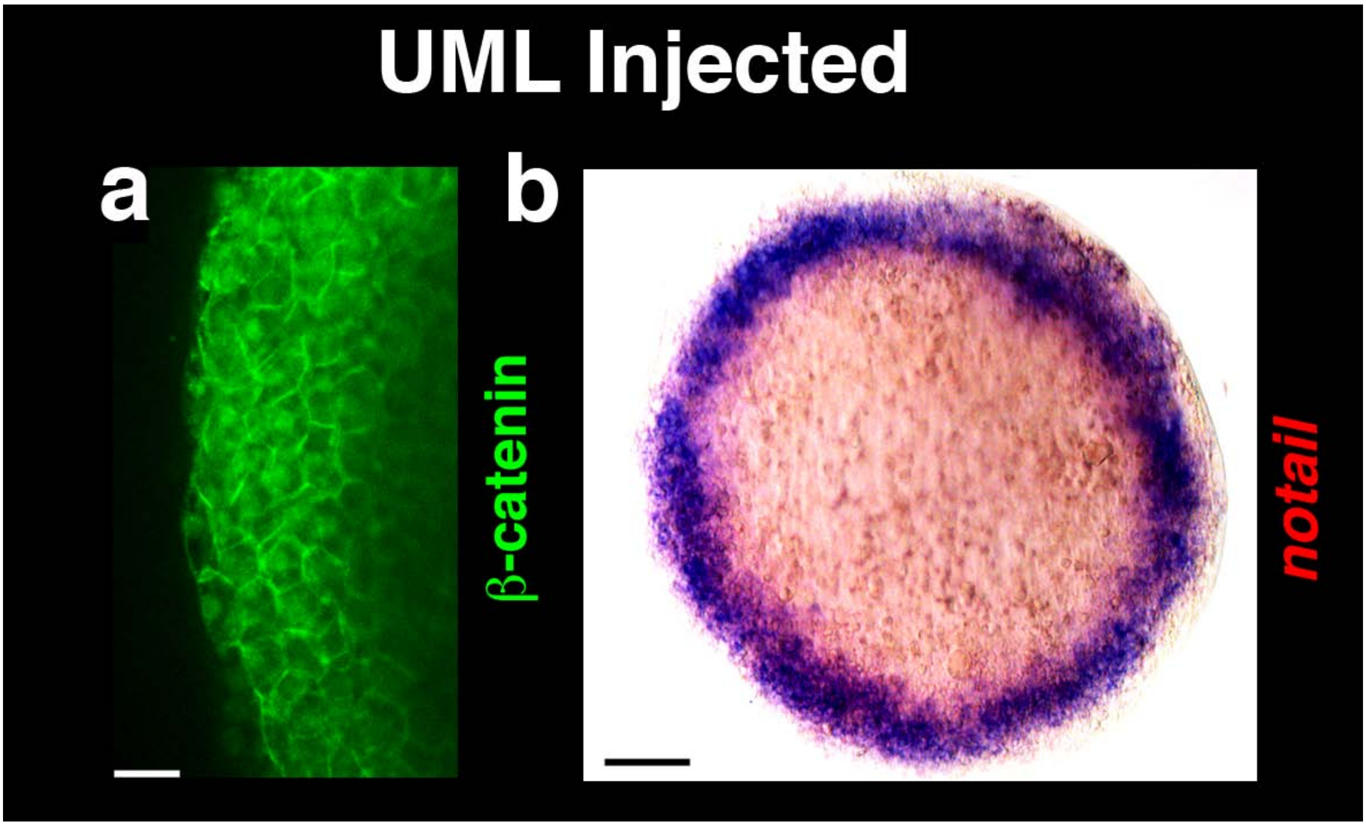

Supplementary Fig. S9

No effect of injection of ultra-magnetic liposomes encapsulating superparamagnetic nano-particles on β-cat nuclear translocation and *ntl* expressing in the absence of magnetic field (**a**) Embryos show both the β-cat nuclear translocation (all, n=7) (**b**) and the *ntl* expression after the injection of ultra-magnetic liposomes at early stage 1 cell, from dome to 50% epiboly stages (all, n=6). This allows the use of magnetic forces to rescue and mimic the dome shape change initiating the epiboly movement in blebbistatin treated embryos to test for β-cat nuclear translocation and *ntl* rescue. The white bar is 20 microns and the black bar is 100 microns. Experiments were replicated 2 times.

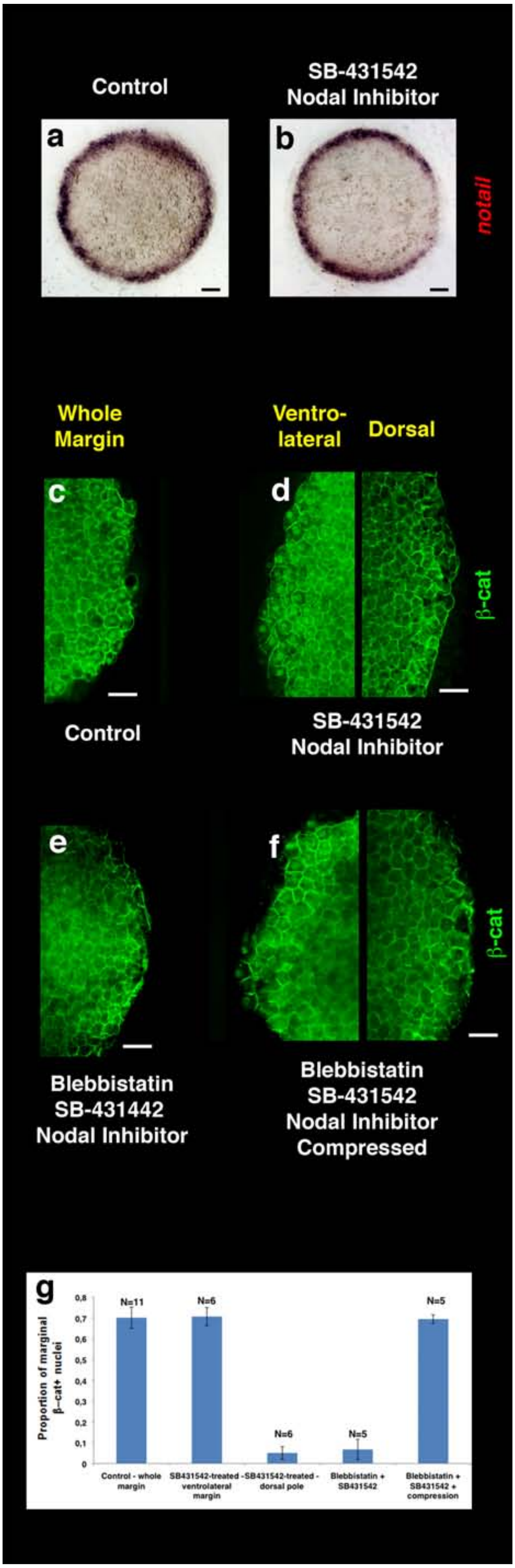

Supplementary Figure S10

Nodal activity is required for nuclear β-catenin translocation and *notail* expression at the dorsal pole, but dispensable in the rest of the marginal zone (**a**) *notail in situ* hybridization signal at dome stage in a control embryo (**b**) and a sibling treated with the Nodal inhibitor SB-451342. (**c**) β-catenin immunostaining signal at dome stage, control. (**d**) SB45-treated sibling, showing loss of nuclear β-catenin translocation at the dorsal pole, and maintenance of nuclear β-catenin around the ventrolateral margin. (**e**) SB45-blebbistatin double-treated embryo, showing loss of nuclear β-catenin all around the margin. Note the relatively noisy effect of double treatment with SB-45 and blebbistatin on labelling. (**f**) Compressed SB45-blebbistatin double-treated sibling, showing rescue of nuclear β-catenin around the ventrolateral margin, but not at the dorsal pole. Note that embryos did not resist *in situ* labelling in addition to blebbistatin, SB-45 and compression treatment, preventing the observation of *ntl* expression within these conditions. (**g**) Quantification of the results. Note that the β-catenin-positive nuclei in compressed SB45-blebbistatin double embryos have been quantified around the ventrolateral margin, excluding the dorsal pole. $p<10^{-6}$ by Student's *t*-test for the comparison between the dorsal pole of treated individuals and their ventrolateral margin; for the comparison between the dorsal pole of treated individuals and the margin of control siblings; for the comparison between the ventrolateral margin of compressed double-treated embryos and the margin of uncompressed double-treated siblings. Error bars are standard deviation. Black bars are 100 microns and white bars are 20 microns.

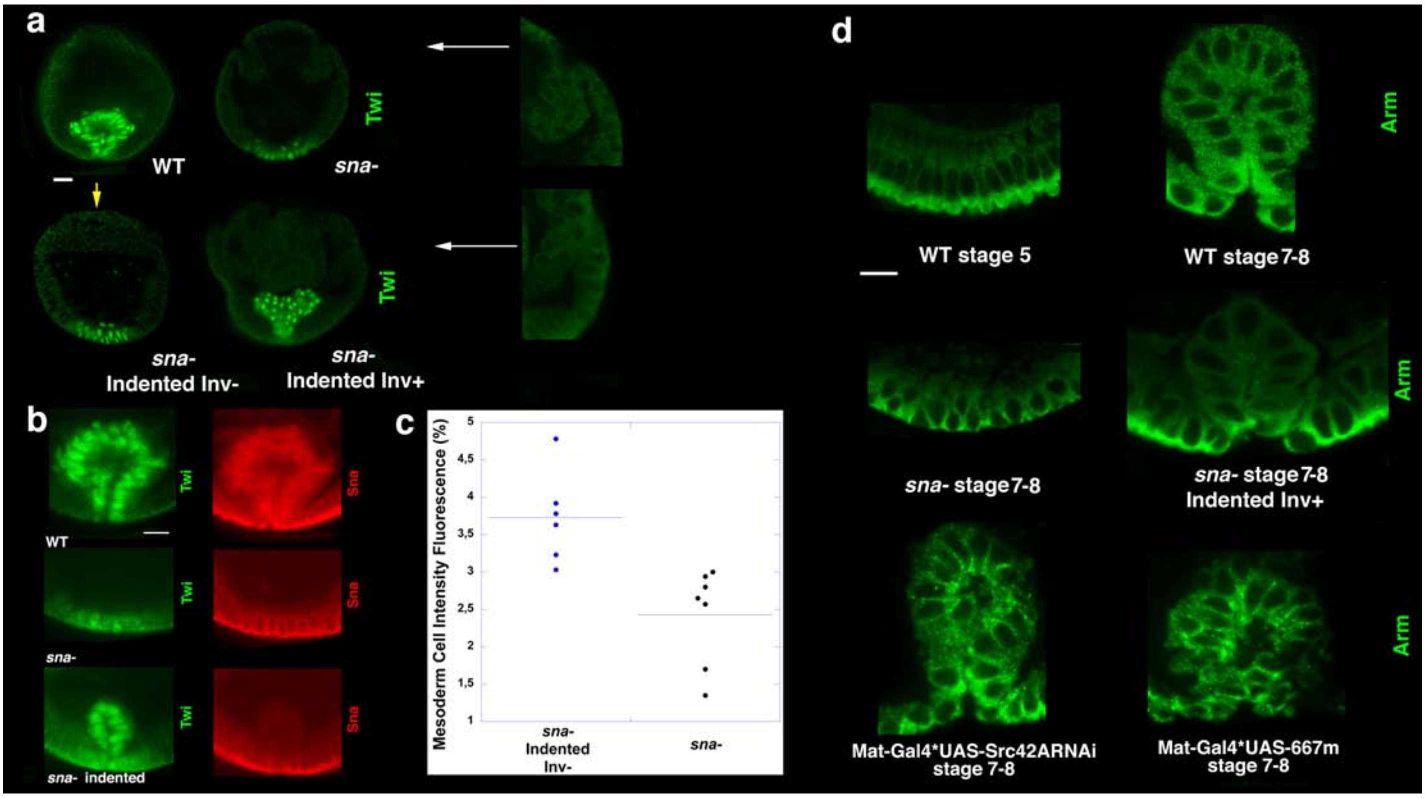

Supplementary Fig. S11

***A posteriori* validation of *Drosophila sna*-/- mutant genotypes and demonstration of mechanically induced Armadillo junctional release and Twi induction during ventral furrow invagination** (**a**) Twi expression is severely decreased in *sna*-/- mutants and is rescued upon invagination, either partially in non-invaginating individuals or up to wild-type levels in invaginating individuals (see Fig. 5e for quantitative analysis and statistics). The yellow arrow shows germ band extension indicating stage 8. Note that in *sna*-/- mutants, invaginated or not, ectopic profound dorso-lateral folds can be observed at stage 8 (white arrows zooms, as described in [70]). (**b**) Double labelling with Snail confirming the *sna* mutant genotype (note that Snail expression is relatively low in the mesoderm at stage 8 [69]). The white bar is 20 microns. (**c**) Indented non-invaginated embryos present a partial but significant, 1.5-fold, rescue of Twi expression (Fig 5e), showing that the high level of Twist expression in invaginated rescued embryos is due to the morphogenetic movement of mesoderm invagination. Note that the distributions in non-indented and indented non-invaginated mutants are non-overlapping, leading to a $p<0.001$ value using the t-student test. Experiments were replicated 2 times. (**d**) Arm immunostaining at 2%FA shows that Arm is mostly localised to apical junctional complexes in presumptive mesoderm in stage 5 in wild-type embryos (n=9). Junctional to cytoplasmic release of Arm is observed in mesoderm concomitantly with invagination at the end of stage 7 - stage 8 (n=12). In *sna*-/- mutants, Arm remains apically localized in the non-invaginating mesoderm (n=8). Invagination rescue upon indentation in *sna*-/- embryos restablishes Arm apical release, with less cytoplasmic Armadillo than in the wild type (n=5). Arm remain apical junctional in both Mat-Gal4*UAS-Src42ARNAi (n=8/9) and Mat-Gal4*UAS-667m (n=9/10) at stage7-8 (see also ventral views of Supplementary Fig. S15a). Note that with the 2% FA classical fixation method used here (see Materials and Methods), hardly detectable low levels of Armadillo may remain in the junctions in the invaginating mesoderm, that can be observed by using the heat MeOH rapid fixation [71] known to extract most of the cytoplasmic Arm that consequently strongly enhances junction labelling compared to cytoplasmic labelling[68], especially using peroxidase amplification. Note also that the 2%FA procedure also makes more diffuse apical junction labelling, and may also remain too high to detect any nuclear Arm (normally detectable at 1% FA –see M, for which cuts were impossible). The epistatis Mat-Gal4*UAS-667m snaRNAi injected experiment was tested, but technically did not support injection perforation followed by indent of the glued embryo, at the onset of the fixative procedure. All experiments were replicated 2 times. The white bars are 10 microns.

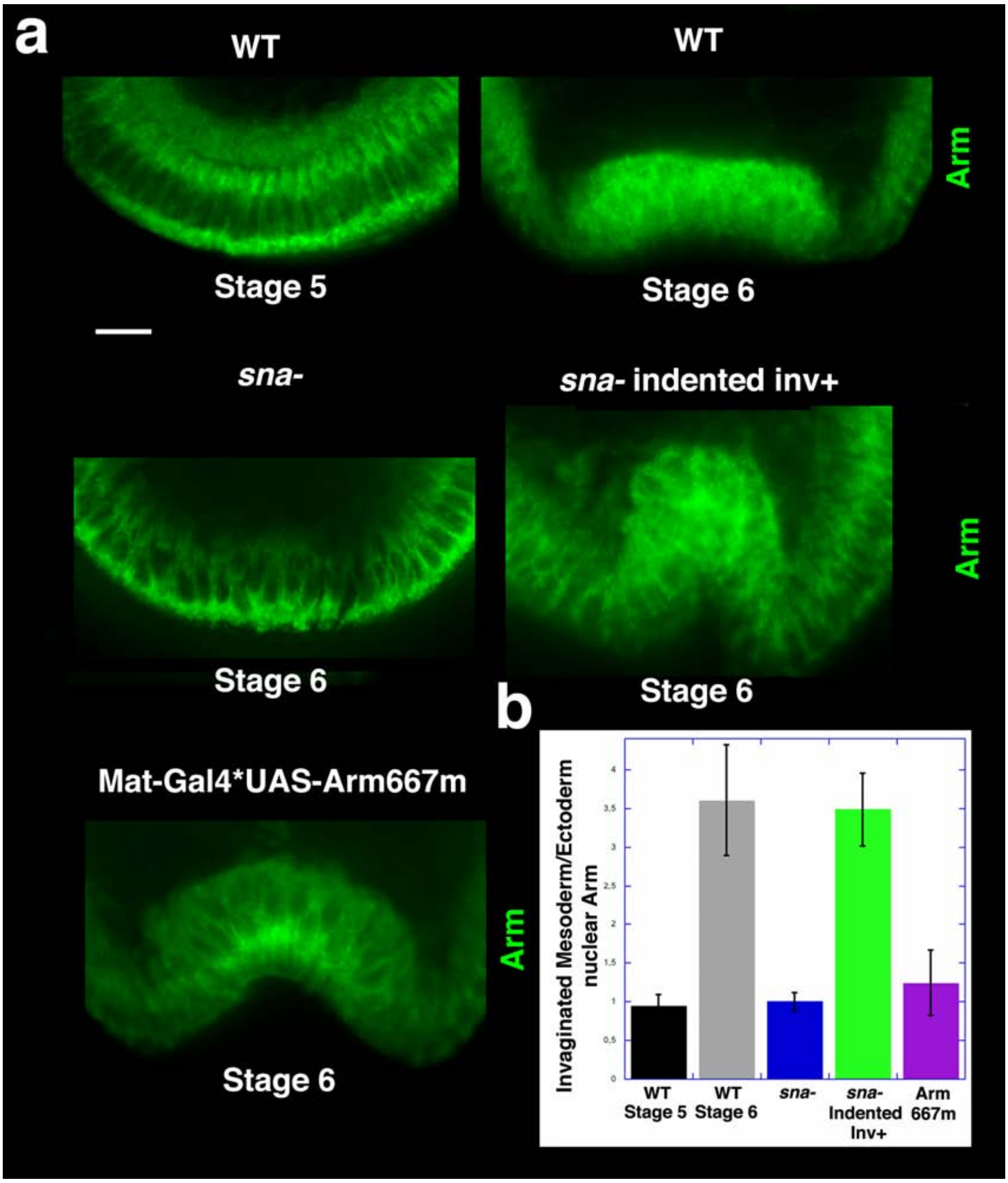

Supplementary Fig. S12

Nuclear translocation of Arm in ArmY66-wild type conditions observed with *20 objective

(**a**) Arm release from the apical membranes to the nuclei observed after 1%FA fixation with *20 air objective, during mesoderm invagination at stage 6 compared to stage 5, is lost in stage 6 *sna* mutants and rescued in mesoderm invaginated *sna* mutants after indent, and is defective in Mat-Gal4*Arm667m Y667 un-phosphorylable Arm mutants. Note the diffuse texture of the image associated with 1%FA procedure, for which nuclear detection of Arm is possible (see M). Higher junctional resolution is achieved with the 2%FA procedure, but characterizes membrane and cytoplasm only (Fig S11d). Note also that in contrast to zebrafish, signalling Armadillo is known to be at best both nuclear and cytoplasmic (see M). (**b**) Histogram: Nuclear enrichment in invaginated mesoderm cells compared to ectoderm quantification. Here error bars are standard error. Number of cases are: WT stage 5 (n=6), WT stage 6 (n=9), *sna* mutants (n=6), rescued in mesoderm invaginated *sna* mutants after indent (n=7), and defective in Mat-Gal4*Arm667m Y667 un-phosphorylable Arm mutants (n=8). All experiments were replicated 2 times.

-

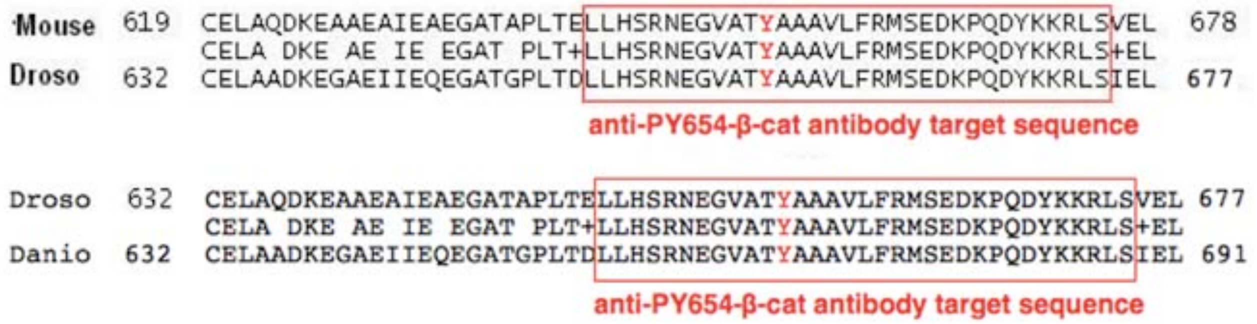

Supplementary Fig. S13

Sequence alignment showing the conservation in Drosophila of the motif recognized by the anti-PY654-β-cat antibody designed against the mouse sequence (**Upper**). Zebrafish β-catenin-1 is 97% identical to mouse β-cat and the sequence is 100% identical in a 100 aminoacid window centred around Drosophila Tyrosine 667 and so to the mouse 654 (see above) (Tyrosine 653 in zebrafish) (**Lower)**. The part of the sequence displayed here is identical for zebrafish β-catenin-1 and β-catenin-2.

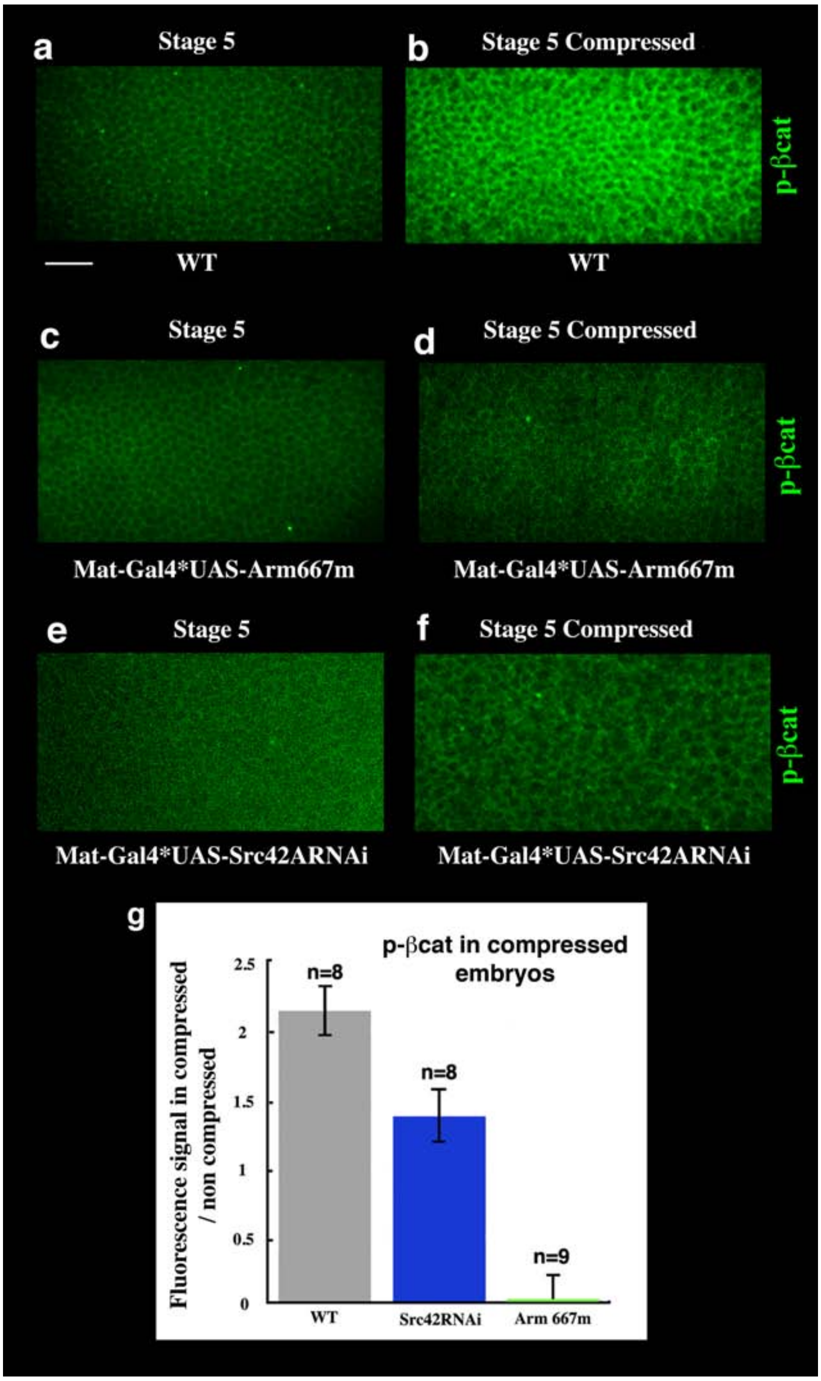

Supplementary Fig. S14

Y667 β-cat phosphorylation is mechanically inducible and requires Src42A activity in *Drosophila* embryos (**a**) PY667-β-cat immunostaining, revealing low junctional and cytoplasmic signal in control stage 5 *Drosophila* embryos. (**b**) β-cat Y667 phosphorylation increases dramatically upon global compression as assessed by immunofluorescence. This response is abolished in (**c,d**) compressed Mat-Gal4*UAS-Arm667m and (e,f) compressed Mat-Gal4*UAS-Src42A-RNAi embryos. (**g**) Quantification of the fluorescent intensity signal of pY667-β-cat in stage 5 globally compressed embryos normalised by the signal of non compressed embryos in the WT (n=8), Src42A RNAi (n=8) and Arm667 (n=9). P<0.01 using Mann-Whitney's exact test. Error bars are standard errors. All experiments were replicated 2 times. The white bar is 20 microns.

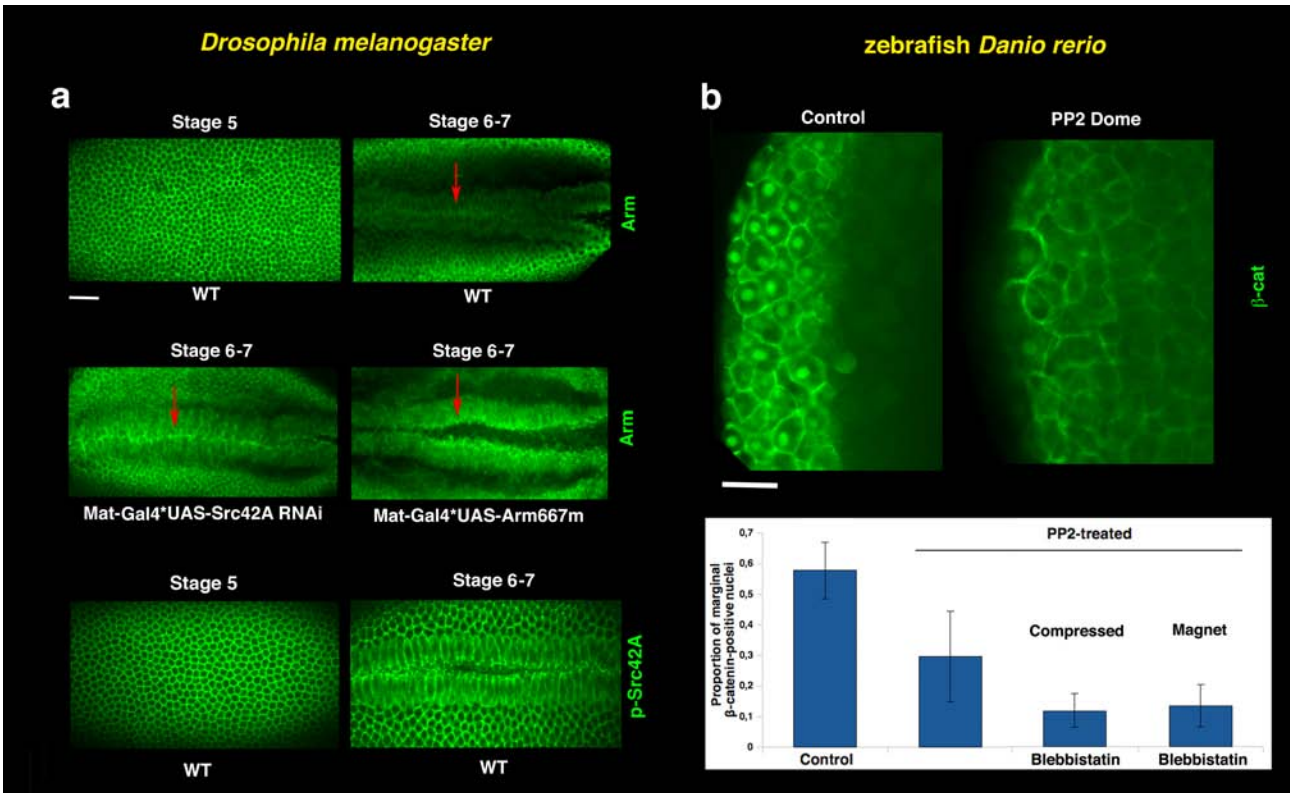

Supplementary Fig. S15

Src-family kinases are required for β-cat junctional release and nuclear translocation (**a**) Arm is predominantly apical junctional in the presumptive mesoderm of wild-type stage 5 *Drosophila* embryos (n=5/5) and gets released from the junctions upon invagination of the ventral furrow at the end of stage 6 – stage 7 (n=5/7) – red arrows, see also Supplementary Fig S11d. Arm remains apical junctional in the ventral furrow of MatGal4*UAS-Src42A-RNAi (n=6/7) and Mat-Gal4*UAS-Arm667m embryos (n=8/10) – red arrows, showing that Y667 phosphorylation of wild type endogenous Arm by Src42A is required for apical junctional release into the mesoderm at the end of stage 6 - stage 7. This is consistent with the increase of turn-over observed in a truncated form of Arm with a mutation on Y667 resembling phosphorylation (additionally lacking the C-terminal 163 aminoacids) in the embryo ectoderm at stage 8 [51]. Finally, Src42A was already activated (phosphorylated) before mesoderm invagination (stage 5, ratio mesoderm/ectoderm R= 0.92+-0.17, n=9), and not over activated at mesoderm invagination stage (stage 7, R=1.05+-0.11, n= 8), with no significant increase of overall mesoderm + ectoderm signals compared to the background (stage 5: 1.81+-0.34, stage 7 1.85+-0.2), confirming Src42A as permissive but not directly involved in the mechanotransduction process [13]. The white bar is 20 microns. (**b**) Marginal nuclear translocation of β-cat is abolished in dome stage zebrafish embryos treated with the Src-family kinase inhibitor PP2. Number of cases are: control (n=12), PP2 treated (n=10), PP2 and blebbistatin treated compressed (n=7) and PP2 blebbistatin compressed with epiboly rescued with magnet (n=5). p=<0.01 according to Mann-Whitney's exact test. Error bars are standard deviation. All experiments were replicated 2 times. The white bar is 20 microns.

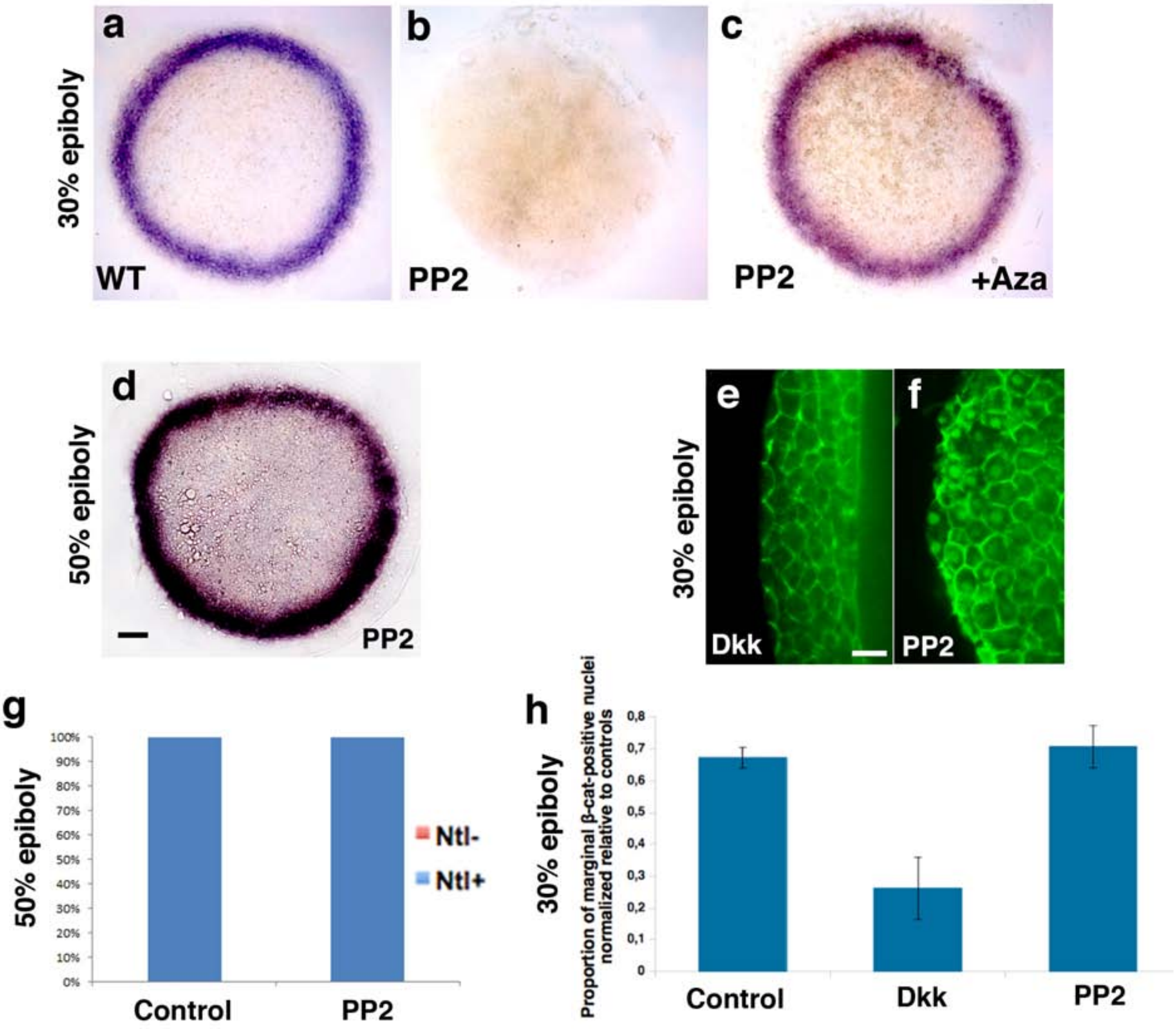

Supplementary Fig. S16

Wnt independent β-cat dependent mechanical signalling controls *notail* expression at the onset of epiboly, but not at later stages of epiboly (**a**) Expression of *notail* at 30% epiboly (**b**) is impaired by PP2 treatment (**c**), and rescued by the Aza GSK-3 inhibitor, in agreement with a β-cat dependent mechanical induction of *notail* at the onset of epiboly (Fig. 1 and Fig. 2). Here the 1 hour 1-azakenpaullone GSK3 inhibitor treatment generating β-cat cytoplasmic accumulation and nuclear translocation led to *ntl* expression in PP2 treated embryos inhibiting pY667- β-cat phosphorylation and its junctional release. Quantitative analysis of (**a,b,c**) are in Fig. 7o. (**d**) PP2 does not prevent *notail* expression at 50% epiboly and after (**e**) as well as β-cat nuclear translocation at 30% epiboly (**f**) whereas Dkk does[25] and Fig. S2b,c). (**g**) Proportion of embryos showing *notail* expression at 50% epiboly in the controls (n=23) and in PP2 treated embryos (n=16). (**h**) Proportion of marginal β-cat positive nuclei at 30% epiboly in controls (n=9), in Dkk injected embryos (n=11) and in PP2 treated embryos (n=11). This shows no role of mechanotransduction but a role of Wnt signalling in the β-cat dependent expression of *notail* at 50% epiboly, in contrast to β-cat dependent mechanical induction initiating *notail* expression at 30% epiboly. $p=<0.001$ according to Mann-Whitney's exact test. Error bars are standard deviation. All experiments were replicated at least 2 times. The black bar is 100 microns and the white bar is 20 microns.

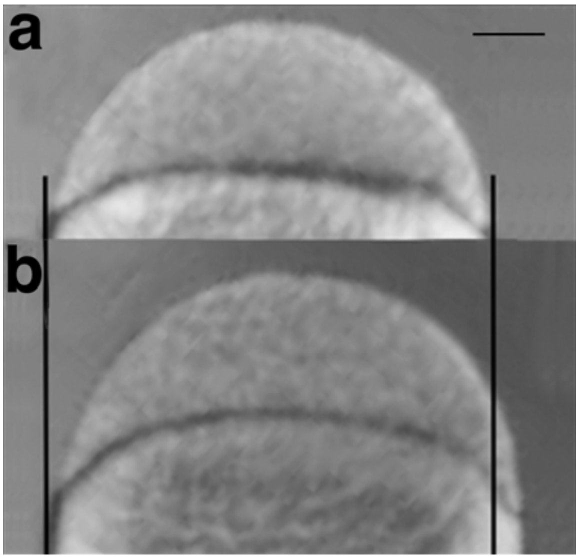

Supplementary Figure S17
Global deformation of blebbistatin treated embryos in response to uni-axial compression
(**a**) Blebbistatin treated embryos at 4.8hpf (i.e equivalent to 30% epiboly in non treated embryos) blocked at sphere phenotypes after blebbistatin treatments. (**b**) Blebbistatin treated embryos deformation at 4.8hpf in response to uni-axial global compression. The black bar is 100 microns.

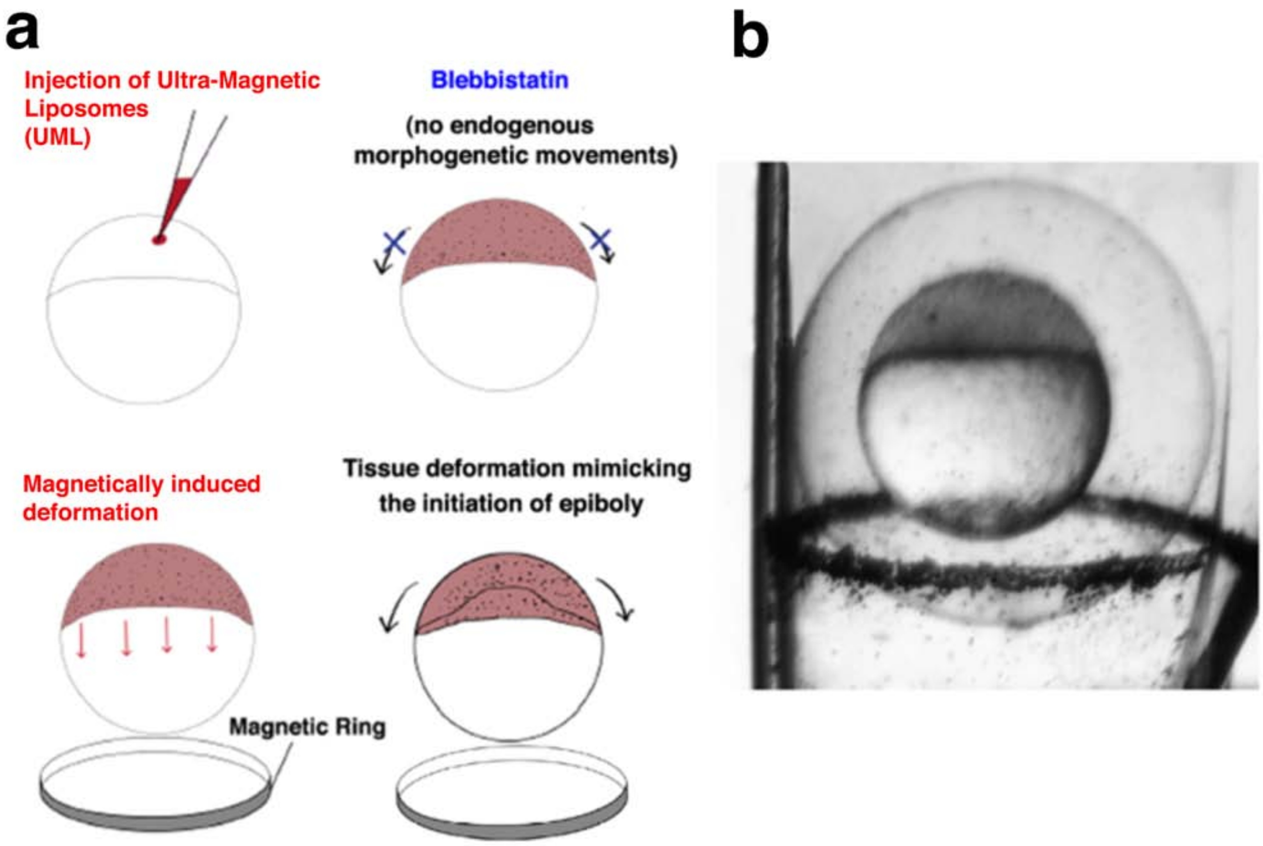

Supplementary Figure S18
Application of a magnetic force by a ring micromagnet on ultra-magnetic liposomes (UML) injected into zebrafish embryos (**a**) Scheme of the experimental sequence, from UML injection, to blebbistatin treatment, to magnetic field application to mimick the epiboly onset mimicking. (**b**) Zebrafish embryo at time zero of the magnetic field application.

Supplementary Methods

Zebrafish embryo fixation and staining

Zebrafish embryos were fixed in 4% paraformaldehyde overnight at 4°C and subsequently dechorionated before storage at -20°C in methanol. β-cat stainings were performed in PBS, 10 % bovine serum albumine, 1 % DMSO after a 10 minute permeabilization in distilled water. In situ hybridizations were performed according to Thisse and Thisse [72] (fixation overnight in 4% PFA PBS 0.1% Tween (PBT), methanol storage, stepwise rehydration in 25%/50%/75%/100% PBT by 5-minutes steps, 1 hour prehybridization and overnight hybridization in hybridization solution at 70°C, probe washing (2x30 minutes (in 50% HB/50% 2xSSC), 1x15 minutes in 2xSSC, 2x30 minutes in 0.2xSSC), followed by probe detection with anti-digoxygenin-alkaline phosphatase antibody (see immunostaining protcol) and NBT/BCIP precipitation staining) and immunostainings according to Schulte-Merker et al. [73] (fixation, storage and rehydration as in the in situ hybridization protocol, followed by 15 minutes permeabilisation in distilled water, 1 hour blocking at room temperature in 10% beef serum 1% DMSO, overnight incubation in blocking solution with 1:250 anti-b-catenin or anti-PY654-b-catenin antibody, 6x20 minutes washes in PBT, blocking and incubation in 1:250 secondary antibody, 6x20 minutes in PBT, mounting in Vectashield and imaging). Mouse anti-beta-catenin antibodies were purchased from Sigma Aldrich (C2206, dilution 1:250) and detected by Alexa 488-anti-mouse antibodies purchased from Molecular Probes. Anti-PY654-beta-catenin antibodies were purchased from Abcam (dilution 1:50). and detected using Cy3-anti-mouse antibodies purchased from Jackson (dilution 1/500).

Double labelling for the *ntl* mRNA and β-catenin were realized by modifying the in situ

hybridization protocol in the following way: the anti-β-catenin antibody was included in the primary antibody solution together with the anti-Dig-AP antibody. After revelation of the NBT/BCIP *ntl* in situ signal, embryos were transferred to the blocking solution and then to the secondary antibody solution according to the immunostaining protocol. After washing, embryos were gradually transferred from PBS 0.1% Tween to TDE (following a 25%, 50%, 75%, 100% dilution series) and finally mounted and imaged in TDE (2,2'-thiodiethanol, purchased from Sigma Aldrich) using a Leica SPE confocal microscope, taking advantage of the far red fluorescence of the NBT/BCIP precipitate (excitation with a 635 nm laser and detection between 750 and 800 nm).

RT-qPCR

RNA was extracted from zebrafish embryos using Trizol purchased from Invitrogen. Reverse transcription was performed using the Agilent AccuScript High Fidelity 1st Strand cDNA synthesis kit. GADPH was used as a reference. The primer sequences were: for *ntl*, forward primer: 5-CTCGGTCCTGCTGGATTTTG-3; for *ntl*, reverse primer: 5-TGTGGTCTGGGACTTCCTTGT-3; for GAPDH, forward primer: 5-GTGTAGGCGTGGACTGTGGT-3; for GAPDH, revers primer: 5-TGGGAGTCAACCAGGACAAATA-3.

Supplementary References

71 Kolsch, V., Seher, T., Fernandez-Ballester, G. J., Serrano, L. & Leptin, M. Control of Drosophila gastrulation by apical localization of adherens junctions and RhoGEF2. *Science* **315**, 384-386 (2007).

72 Thisse, C. & Thisse, B. High-resolution in situ hybridization to whole-mount zebrafish embryos. *Nat Protoc* **3**, 59-69, doi:10.1038/nprot.2007.514 (2008).

73 Schulte-Merker, S., Ho, R. K., Herrmann, B. G. & Nusslein-Volhard, C. The protein product of the zebrafish homologue of the mouse T gene is expressed in nuclei of the germ ring and the notochord of the early embryo. *Development* **116**, 1021-1032 (1992).
